# Baicalin alleviates APEC-induced ileal barrier injury in broiler chickens by modulating inflammatory responses, microbiota composition, and neuro-immune-related transcriptional profiles

**DOI:** 10.3389/fcimb.2026.1967613

**Published:** 2026-10-20

**Authors:** Weimei Liu, Xiaodan Liu, Jiameng Li, Qingjie Zhang, Jianhong Li, Hongyu Wang, Xu Ji, Shenghe Li, Feng Zhang

**Affiliations:** 1 College of Animal Science, Anhui Science and Technology University, Fengyang, Anhui, China; 2 College of Life Sciences and Technology, Northeast Agricultural University, Harbin, Heilongjiang, China; 3 Anhui Province Key Laboratory of Animal Nutrition Regulation and Health, Anhui Science and Technology University, Fengyang, Anhui, China; 4 Anhui Province Key Laboratory of Livestock and Poultry Product Safety Engineering, Institute of Animal Science and Veterinary Medicine, Anhui Academy of Agricultural Sciences, Hefei, Anhui, China

**Keywords:** avian pathogenic *Escherichia coli*, baicalin, broiler chickens, ileal barrier injury, ileal microbiota, neuro–immune signaling

## Abstract

**Introduction:**

Avian pathogenic *Escherichia coli* (APEC) can disrupt intestinal barrier integrity and induce mucosal inflammation in broiler chickens. Baicalin, a flavonoid derived from *Scutellaria baicalensis*, has anti-inflammatory and intestinal protective properties, but its effects on APEC-associated ileal injury and the accompanying microbial and host transcriptional responses remain incompletely understood.

**Methods:**

A total of 288 one-day-old broilers were assigned to four treatments: a basal diet with saline administration (BD+Saline), a basal diet with APEC challenge (BD+APEC), and basal diets supplemented with 150 (LB+APEC) or 300 mg/kg baicalin (HB+APEC) followed by APEC challenge. Birds were orally challenged with APEC O78 on d 18–20 and again on d 39–41, with samples collected on d 21 and d 42, respectively. Ileal morphology, goblet cell numbers, serum permeability-related markers, barrier-, inflammatory-, and Nrf2-related gene expression, ileal microbiota, and transcriptomic profiles were evaluated.

**Results:**

APEC challenge impaired ileal morphology and reduced goblet cell numbers at both sampling ages, with increased serum DAO and LPS following the second challenge. Baicalin improved villus architecture, goblet cell abundance, tight junction-related gene expression, and inflammatory responses, and reduced serum DAO and LPS following the first challenge. It also attenuated APEC-associated upregulation of Nrf2-related antioxidant genes. Microbiota analysis showed increased *Corynebacterium* at both sampling ages and *Weissella* following the first challenge. Transcriptomic and qRT-PCR analyses highlighted *NPY*, *NPY1R*, *AGTR1*, *IL-10*, *IFN-γ*, and *MYL10* as genes associated with neuro-immune-, cytokine-, and cytoskeleton-related responses.

**Conclusion:**

Dietary baicalin alleviated APEC-induced ileal barrier injury in broiler chickens by improving mucosal structure, goblet cell abundance, tight junction-related gene expression, and inflammatory responses. Baicalin also altered the ileal microbiota, including increased relative abundances of *Corynebacterium* and *Weissella*, and modified neuro-immune-related transcriptional responses involving *NPY*, *NPY1R*, *AGTR1*, *IL-10*, *IFN-γ*, and *MYL10*. Collectively, these findings demonstrate the beneficial effects of dietary baicalin on intestinal barrier integrity, inflammation, and microbial and transcriptional responses during APEC challenge, supporting its potential as a plant-derived functional feed additive for improving intestinal health in broilers.

## 1 Introduction

The global surge in intensive poultry operations has closely paralleled the escalating prevalence of avian pathogenic *Escherichia coli* (APEC) infections on a global scale (Huang et al., 2025). The pervasive global expansion of antimicrobial-resistant variants has compromised the effectiveness of standard antimicrobial therapies within avian production disease prevention and control, exacerbating avian morbidity and mortality, and economic losses, thereby threatening production efficiency and the sustainable development of the avian production sector (Abreu et al., 2023; Tan et al., 2023). APEC is an important bacterial pathogen in the poultry industry and can cause various colibacillosis diseases, such as airsacculitis, peritonitis, such systemic conditions drastically impair avian health, resulting in a sharp decline in broiler growth efficiency and performance (Li et al., 2025). In addition to classical systemic infection, accumulating evidence consistently indicates that APEC can also directly act on the intestine, induce local inflammatory responses, compromise the mucosal architecture epithelial structure, and impair mucosal integrity and gut barrier performance, thereby increasing the risk of bacterial translocation and secondary infection (Ye et al., 2025; Zha et al., 2025). Intestinal barrier damage not only reduces the host defense capacity against pathogenic stimulation, but also further aggravates inflammation, oxidative stress, and microecological imbalance, ultimately affecting nutrient absorption and intestinal health in broiler chickens (Cheng et al., 2022; Li et al., 2024; Wan et al., 2024). Therefore, in the context of restricted antibiotic use and continuously increasing drug resistance pressure, the development of plant-derived nutritional intervention strategies that can alleviate APEC-related intestinal injury has become an important direction in poultry nutrition and health research.

Derived as the primary bioactive flavone from *Scutellaria baicalensis*, baicalin exhibits potent anti-inflammatory properties, radical-scavenging capacities, and mucosal-stabilizing benefits (Wen et al., 2023). Extensive literature demonstrates that gut lesions is attenuated and mucosal shield integrity is optimized by baicalin via reducing oxidative stress, inhibiting the liberation of pro-inflammatory mediators, via modulating host immunity and stabilizing the homeostatic equilibrium of the intestinal microenvironment microbiome (Hu et al., 2021; Gao et al., 2024; Liang M. et al., 2024). Its intestinal protective effect is related not only to its own biological activity, but also closely to its metabolic transformation in the intestine (Liu et al., 2024a; Liu X. et al., 2025). With the participation of the intestinal microbiota, baicalin can be transformed into active metabolites such as baicalein, which further act on intestinal epithelial cells and exert local anti-inflammatory, antibacterial, and barrier-protective effects (Gao et al., 2024). In addition, baicalin can also alleviate tissue injury by regulating inflammation-related intracellular cascades including TLR4/NF-κB, and has shown effects in improving intestinal injury and maintaining barrier homeostasis in bacterial infection and inflammation-related intestinal injury models (Cheng et al., 2017; Hu et al., 2021; Zhu et al., 2026). In the APEC infection model relatively close to the present study, prior research has suggested that baicalin can alleviate intestinal injury and improve the local inflammatory status (Cheng et al., 2022; Cao et al., 2025). However, the specific mechanism by which baicalin alleviates APEC-induced intestinal barrier injury remains unclear, especially whether it exerts synergistic protective effects by regulating intestinal microbiota homeostasis, the expression of barrier-related molecules, and related transcriptional regulatory networks, However, few studies have integrated intestinal morphology, barrier-related gene expression, ileal microbiota, and transcriptomic responses to clarify how baicalin modulates APEC-induced ileal barrier injury in broilers.

Consequently, this trial characterized the bioactivity of baicalin on APEC-induced ileal barrier injury in broiler chickens by evaluating intestinal morphology, barrier-related indicators, inflammatory and antioxidant responses, ileal microbiota, and transcriptomic changes at two growth stages. The results may provide evidence for understanding the potential microbiota-host transcriptional mechanisms involved in the protective effects of baicalin.

## 2 Materials and methods

### 2.1 Animal ethics statement

Every avian trial followed the ARRIVE guidelines and was sanctioned by the Institutional Animal Ethics Committee of Anhui Science and Technology University (Permit No. AHSTU2025007). All experimental operations strictly conformed to university regulations alongside national frameworks established for the humanitarian care of laboratory animals.

### 2.2 Bacterial strain and baicalin

The APEC O78 strain, originally isolated from a diseased chicken, was kindly provided by the Animal Husbandry and Veterinary Research Department, Anhui Academy of Agricultural Sciences (Hefei, Anhui, China). The strain was recovered on MacConkey agar at 37 °C and subsequently cultured in nutrient broth for 16 h. After culture, the bacterial cells were harvested, washed with sterile saline, and resuspended in sterile saline. Bacterial growth was monitored at OD600, and the resulting bacterial suspension was adjusted to 1 × 10^8^ CFU/mL before oral challenge. Thus, nutrient broth was used for bacterial propagation, whereas sterile saline served as the final vehicle for oral inoculation. Baicalin (purity ≥ 90%) was purchased from Macklin Biochemical Technology Co., Ltd. (Shanghai, China).

### 2.3 Experimental design

A total of 288 healthy 1-d-old Arbor Acres (AA) broilers were randomly assigned to four treatments using a completely randomized design, with six replicate pens per treatment and 12 birds per pen (equal numbers of males and females). Birds were housed in separate floor pens with ad libitum access to feed and water. The initial room temperature was maintained at 35 °C and gradually reduced by 3 °C per week to 26 °C, with relative humidity maintained at 50%–60%. Birds were immunized according to routine procedures. The four treatments were as follows (1): BD+Saline, birds fed the basal diet and orally administered saline (2); BD+APEC, birds fed the basal diet and orally challenged with APEC (3); LB+APEC, birds fed the basal diet supplemented with 150 mg/kg baicalin and orally challenged with APEC; and (4) HB+APEC, birds fed the basal diet supplemented with 300 mg/kg baicalin and orally challenged with APEC. The experimental diets were provided from d 1 to 42, with the first 3 d considered an adaptation period. The experiment included two oral APEC challenge periods. Birds in the APEC-challenged groups received 3 mL of APEC O78 suspension (1 × 10^8 CFU/mL) by oral gavage once daily on d 18–20 and again on d 39–41. The oral route was selected because the present study focused on intestinal responses and has also been used in previous APEC O78 challenge models to approximate natural exposure (Helmy et al., 2023). The repeated challenge schedule was established based on preliminary experiments to induce measurable intestinal responses while avoiding the high mortality observed with parenteral challenge. Birds in the BD+Saline group received an equal volume of sterile saline by oral gavage following the same schedule. The first and second challenge periods preceded sampling on d 21 and d 42, respectively; therefore, the d 42 sampling represented the response following the second APEC challenge rather than a delayed response to the first challenge. The overall experimental design is shown in **Figure 1**. The basal diets were formulated according to the Chinese Broiler Feeding Standard (NY/T 33-2004), and their ingredient composition and calculated nutrient levels are shown in **Supplementary Table S1**.

**Figure 1:**
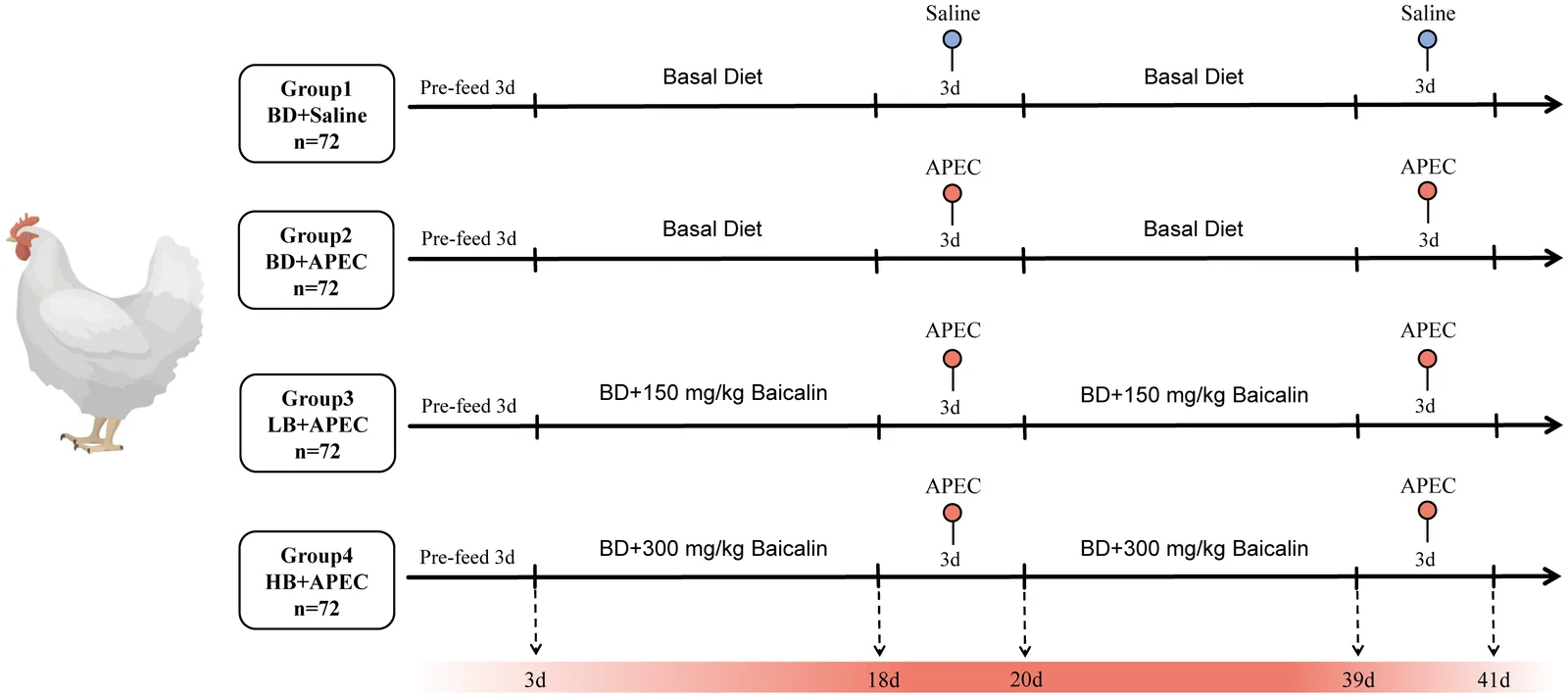
Design of the animal experiment. BD, basal diet; APEC, avian pathogenic *Escherichia coli*; LB, basal diet supplemented with 150 mg/kg baicalin; HB, basal diet supplemented with 300 mg/kg baicalin; Saline, sterile saline.

### 2.4 Sample collection

On d 21 and d 42, the chickens were weighed by replicate, feed consumption for each replicate was documented. Average daily gain (ADG), average daily feed intake (ADFI), and the feed-to-gain ratio (F: G) were calculated for d 1–21, d 22–42, and d 1–42. F: G was calculated as feed intake divided by body weight gain. On d 21 and d 42, three birds were selected from each pen for sample collection, resulting in 18 birds per treatment at each sampling age (3 birds/pen × 6 pens/treatment). The birds sampled on d 21 and d 42 were different individuals. Before sampling, birds were fasted for 12 h. Blood was collected from the wing vein and centrifuged at 3,000 × g for 15 min at 4 °C. Serum was collected and stored at −80 °C until analysis. The birds were then euthanized by cervical dislocation, and ileal samples were collected immediately for subsequent analyses. The ileum was collected immediately after euthanasia. A 1-cm segment from the mid-ileum was fixed in 4% paraformaldehyde for histological analysis. Adjacent ileal tissue from the same anatomical region was rinsed with ice-cold PBS, snap-frozen in liquid nitrogen, and stored at −80 °C for subsequent qRT-PCR and transcriptomic analyses. Distal ileal digesta were collected separately for microbiota analysis.

### 2.5 Determination of serum DAO, D-lactic acid, and LPS levels

Serum diamine oxidase (DAO; Cat. No. DKM9507A), D-lactic acid (D-LA; Cat. No. DKM9437A), and lipopolysaccharide (LPS; Cat. No. DKM5263A) concentrations were measured using commercial ELISA kits (Beijing Dakemei Technology Co., Ltd., Beijing, China) according to the manufacturer’s instructions.

### 2.6 Intestinal morphology analysis

Fixed ileal samples were embedded in paraffin and sectioned. Following deparaffinization, the sections were stained using a High-Definition utilizing an H&E (hematoxylin and eosin) histochemical staining platform supplied by Wuhan Servicebio Technology Co., Ltd. (Wuhan, China). After dehydration and mounting, the sections were subjected to microscopic examination and image acquisition. For each section, ten intact villus-crypt structures were architectural targets for assessing villar altitude and crypt depression through Case Viewer 2.4.

### 2.7 Goblet cell counting

Histochemical identification of ileal goblet cells was achieved through AB-PAS (Alcian blue-periodic acid-Schiff) tracking. For each broiler, ileal goblet cells were quantified across a minimum of ten structurally intact villus-crypt units. Goblet cells were counted separately in ten well-oriented villi and ten crypts per sample, and the average goblet cell density within villus and crypt were calculated, respectively.

### 2.8 Quantitative real-time PCR

Total RNA was extracted from mid-ileal tissue using the RNA Easy Fast Tissue/Cell Kit (TIANGEN, Beijing, China; Cat. No. DP451) according to the manufacturer’s instructions. Genomic DNA was removed, and cDNA was synthesized using the HiScript III 1st Strand cDNA Synthesis Kit (+gDNA eliminator) (Vazyme Biotech Co., Ltd., Nanjing, China; Cat. No. R312). Quantitative real-time PCR was performed using ChamQ SYBR qPCR Master Mix (Vazyme Biotech Co., Ltd., Nanjing, China; Cat. No. Q311-02) on a QuantStudio 5 Real-Time PCR System (Applied Biosystems, USA). Gene-specific primers were designed using Primer 5.0 and synthesized by Sangon Biotech Co., Ltd. (Shanghai, China), and the primer sequences are listed in **Supplementary Table S2**. β-Actin and GAPDH were used as reference genes, and the geometric mean of their Ct values was used for normalization. Relative mRNA expression was calculated using the 2^−ΔΔCt method.

### 2.9 16S rRNA gene sequencing of ileal bacteria

On days 21 and 42, distal ileal chyme was harvested for subsequent bacterial genomic DNA isolation. For 16S rRNA gene profiling, the collected ileal digesta was transferred to Shanghai Ling En Biotechnology Co., Ltd. (Shanghai, China). The main procedures from DNA extraction to library construction and sequencing were as follows. First, isolation of total genomic DNA from ileum via the E.Z.N.A.^®^ Soil DNA Kit (Omega Bio-Tek, Norcross, GA, USA) under the guidance of operating instructions, and the resulting template density was detected by electrophoresis. Second, To Profile luminal microbial ecology, the polymorphic V3–V4 Hypervariable loci of the eubacterial 16S rRNA gene were targeted for amplicon generation. This was executed utilizing the standard primer pair designated as 341F (5’-CCTAYGGGRBGCASCAG-3’) and 806R (5’-GGACTACNNGGGTATCTAAT-3’), subsequently, the library architecture was finalized by utilizing the SMR Tbell™ Template Prep Kit 1.0 platform (Pacific Biosciences). Finally, the amplicon library was amplified according to the standard procedure and subsequently resolved utilizing the PacBio Sequel II sequencing architecture.

SMRTLINK (V9) served to execute quality evaluation and the subsequent filtration of the raw sequences obtained from sequencing. OTU representative sequence Sequences were clustered with a 0.97 similarity index serving as the definitive partition threshold chimeric artifacts were purged from the dataset through UCHIME implementation, subsequent to the primary sequence clustering executed via UPARSE v7.1 (). RDP classifier 2.2 () was used for taxonomic annotation, facilitating the systematic assessment of microbial taxonomic profiles across a full range of phylogenetic hierarchies. Alpha diversity was calculated based on the OTU abundance table, while community dissimilarity across treatments was evaluated via Bray-Curtis matrices and subsequently projected through using Principal Component Analysis (PCA) and Principal Coordinates Analysis (P CoA) of differences in the abundance distribution of species and functions among samples.

### 2.10 Ileal transcriptomic analysis

For eukaryotic transcriptomic profiling, harvesting and processing of the ileal tissue matrices were outsourced to Shanghai Ling En Biotechnology Co., Ltd. (Shanghai, China). The methodical sequence from raw transcript isolation through library preparation and high-throughput sequencing was executed under strict parameters. Initially, total RNA isolation was performed using TRIzol^®^ reagent (Invitrogen) in compliance with the vendor’s specifications, followed by enzymatic degradation of contaminating genomic DNA via DNase I (TaKaRa). Subsample concentrations were subsequently evaluated utilizing an ND-2000 spectrophotometer (Nano Drop) to ensure only pristine templates advanced to downstream sequencing. Next, 1 μg aliquots of the vetted total RNA were converted into sequencing-ready libraries using the Illumina Tru Seq™ RNA Sample Prep Kit (San Diego, CA) framework. Ultimate library quantification was completed via TBS380 fluorometric evaluation before the constructs were subjected to 150-bp paired-end sequencing operations hosted on an Illumina Nova Seq 6000 architecture (150bp×2).

Artifact elimination and sequence refinement of the raw paired-end libraries were executed via Trimmomatic v0.36, which applied a sliding window size of 4 with a minimum quality threshold of 15, while discarding fragments under 75 bp (SLIDINGWINDOW:4:15 MINLEN:75). The resulting high-quality datasets were then directionally mapped back to the reference genome utilizing HISAT2 under its standard configuration. Qualimap v2.2.1 was used to assess alignment quality, and HTSeq was used to count the reads for each gene. Gene expression was quantified as TPM for visualization, whereas raw read counts were used for differential expression analysis using edgeR, with |log2FC| > 1 and FDR < 0.05 as the screening criteria. Goatools and KOBAS were implemented to conduct functional annotation across both Gene Ontology (GO) supplemented by functional pathway mapping against the Kyoto Encyclopedia of Genes and Genomes (KEGG) knowledgebase, applied in a comparative manner across the study groups, with statistical divergence established at a Bonferroni-adjusted threshold of *P* < 0.05. To visualize structural variances across the high-dimensional dataset, we implemented PCA as an unsupervised dimensionality-reduction framework, volcano plots of DEG distribution, and functional annotation analyses were all visualized using R packages.

Based on KEGG pathway analysis, STRING was used core hub nodes were characterized based on topological degree centrality, and genes with the highest degree values in derived from the protein-protein interaction (PPI) infrastructure were targeted for downstream mechanistic evaluation enriched pathways, with a confidence score of 0.7. After network construction, Cytoscape 3.10.3 was used for network analysis, ranking according to degree centrality (Degree), and visualization.

### 2.11 Statistical analysis

Data were checked for normality and homogeneity of variance before statistical analysis. The BD+Saline and BD+APEC groups were compared using an independent-samples t-test to evaluate the effect of APEC challenge. The BD+APEC, LB+APEC, and HB+APEC groups were analyzed by one-way ANOVA followed by Tukey’s test to evaluate the effect of baicalin supplementation under APEC challenge. For microbial community analysis, beta diversity was assessed using Bray–Curtis distances and differences in community structure were evaluated by PERMANOVA. Pearson’s correlation analysis was used to assess associations between variables, with false discovery rate correction applied where appropriate. Data are presented as mean ± SEM, and differences were considered significant at *P* < 0.05. Statistical analyses were performed using SPSS 26.0 and R, and figures were generated using GraphPad Prism 10.0.

## 3 Results

### 3.1 Growth performance

Growth performances including body weight (BW), average daily gain (ADG), average daily feed intake (ADFI), and feed conversion efficiency (F: G) were evaluated for 1–21 d, 22–42 d, and 1–42 d (**Table 1**). From 1 to 21 days of age, compared with the BD+Saline group, the F:G ratio in the BD+APEC group was significantly decreased (*P* < 0.01). From 1 to 42 days of age, ADG showed a numerical decreasing trend during the BD+APEC, LB+APEC, and HB+APEC groups (overall *P* = 0.06). Except for the decreasing trend in ADG in the HB+APEC group during 1–42 d, no significant differences were observed in BW, ADFI, ADG, and F: G among groups.

**Table 1:** Effects of dietary supplementation with baicalin (BA) on the growth performance of broiler chickens.

Measure	Experimental diets				SEM	*p*-Value	
	BD+Saline	BD+APEC	LB+APEC	HB+APEC		Challenge	Baicalin
1d BW, g	47.87	47.25	46.49	47.43	0.25	0.09	0.29
21d BW, g	568.10	613.33	617.03	574.51	12.57	0.19	0.29
42d BW, g	1789.27	1848.66	1830.21	1718.53	27.87	0.19	0.29
1–21 d							
ADG, g/d	24.77	26.96	27.14	25.10	0.60	0.18	0.35
ADFI, g/d	41.93	40.89	43.44	41.22	0.72	0.52	0.33
F: G, g/g	1.69	1.52	1.60	1.64	0.03	<0.01	0.11
22–42 d							
ADG, g/d	58.15	58.83	57.77	54.48	1.05	0.83	0.23
ADFI, g/d	109.02	108.13	110.08	101.74	2.33	0.87	0.35
F: G, g/g	1.88	1.85	1.91	1.87	0.05	0.82	0.70
1–42 d							
ADG, g/d	41.46	42.89	42.45	39.79	0.66	0.47	0.06
ADFI, g/d	69.43	69.40	72.82	68.14	1.14	0.99	0.24
F: G, g/g	1.68	1.62	1.72	1.71	0.04	0.60	0.39

### 3.2 Intestinal permeability

At 21 d, serum DAO, D-LA, and LPS levels did not differ significantly between the BD+Saline and BD+APEC groups (**Table 2**). Compared with the BD+APEC group, serum DAO and LPS levels were significantly decreased in both the LB+APEC and HB+APEC groups (*P* < 0.01), and serum D-LA was significantly decreased in the LB+APEC group (*P* < 0.05). At 42 d, serum DAO and LPS levels were significantly higher in the BD+APEC group than in the BD+Saline group (*P* < 0.01), whereas serum D-LA did not differ significantly between these two groups. Compared with the BD+APEC group, serum D-LA was significantly increased in the HB+APEC group (*P* < 0.01), while DAO and LPS levels were not significantly altered by baicalin supplementation.

**Table 2:** Effects of dietary baicalin supplementation on the intestinal permeability of APEC-challenged broilers.

Measure	Experimental diets				SEM	*p*-Value	
	BD+Saline	BD+APEC	LB+APEC	HB+APEC		Challenge	Baicalin
21 d							
DAO, ng/mL	334.85	332.79^a^	285.36^b^	277.61^b^	7.74	0.91	<0.01
D-LA, μmol/L	161.91	164.21^a^	140.09^b^	150.34^ab^	4.20	0.87	<0.05
LPS, pg/mL	514.10	522.22^a^	463.31^b^	429.51^b^	9.37	0.76	<0.01
42 d							
DAO, ng/mL	476.11	612.31^**^	591.22	609.9	15.75	<0.01	0.78
D-LA, μmol/L	177.47	187.19^b^	216.90^b^	284.70^a^	7.92	0.42	<0.01
LPS, pg/mL	664.40	824.63^**^	768.87	813.57	16.87	<0.01	0.28

Asterisks indicate differences between the BD+Saline and BD+APEC groups based on an independent-samples t-test (*P* < 0.05, *P* < 0.01), whereas different lowercase letters indicate significant differences among the BD+APEC, LB+APEC, and HB+APEC groups based on one-way ANOVA (*P* < 0.05).

### 3.3 Ileal morphology

At d 21, compared with the BD+Saline group, the BD+APEC group showed decreased villus height and V/C (P < 0.01) and increased crypt depth (P < 0.05). Compared with the BD+APEC group, both LB+APEC and HB+APEC increased villus height and V/C (P < 0.01), whereas crypt depth was decreased only in the HB+APEC group (P < 0.05). At d 42, compared with the BD+Saline group, the BD+APEC group showed decreased villus height and V/C and increased crypt depth (P < 0.01). Compared with the BD+APEC group, both LB+APEC and HB+APEC increased villus height and V/C (P < 0.01) and decreased crypt depth. Compared with the LB+APEC group, the HB+APEC group showed greater villus height and crypt depth (P < 0.05) (**Figures 2A, B**).

**Figure 2:**
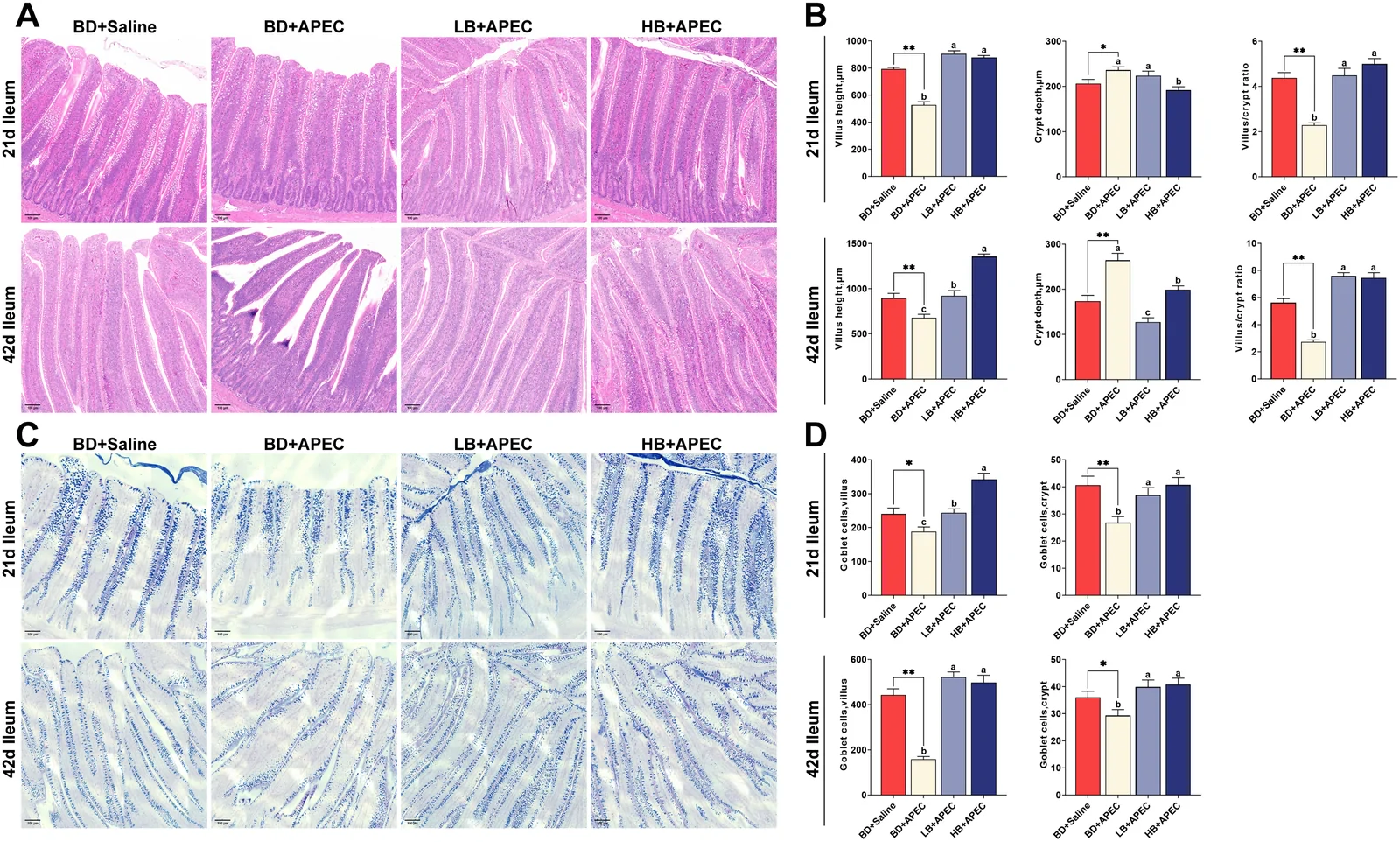
Effects of dietary baicalin supplementation on ileal histomorphology and goblet cells in broilers after avian pathogenic *Escherichia coli* (APEC) challenge. **(A)** Representative images of H&E-stained ileal sections. **(B)** Ileal villus height, crypt depth, and villus height-to-crypt depth ratio (V/C). **(C)** Representative images of AB-PAS-stained ileal sections. **(D)** Goblet cell counts in the ileal villi and crypts. BD+Saline, broilers fed the basal diet and orally administered saline; BD+APEC, broilers fed the basal diet and orally challenged with APEC; LB+APEC, broilers fed the basal diet supplemented with 150 mg/kg baicalin and orally challenged with APEC; HB+APEC, broilers fed the basal diet supplemented with 300 mg/kg baicalin and orally challenged with APEC. *P < 0.05 and **P < 0.01 indicate differences between the BD+Saline and BD+APEC groups; ns indicates no significant difference. Different lowercase letters indicate significant differences among the BD+APEC, LB+APEC, and HB+APEC groups (*P* < 0.05).

### 3.4 Ileal goblet cells

At 21 d, compared with the BD+Saline group, the number of goblet cells in the villi (*P* < 0.05) and crypts (*P* < 0.01) in the BD+APEC group was significantly decreased. Compared with the BD+APEC group, the number of goblet cells in the villi and crypts in the LB+APEC and HB+APEC groups was increased (*P* < 0.05). Among them, the number of goblet cells in the villi in the HB+APEC group was higher than that in the LB+APEC group (*P* < 0.05). At 42 d, the numbers of goblet cells in villi and crypts were significantly lower in the BD+APEC group than in the BD+Saline group (P < 0.05andP < 0.01). Both LB+APEC and HB+APEC significantly increased goblet cell numbers compared with the BD+APEC group (P < 0.01) (**Figures 2C, D**).

### 3.5 Ileal physical barrier

At 21 d, no significant differences in *ZO-1*, *ZO-2*, and *Claudin-1* expression were observed between the BD+Saline and BD+APEC groups. Compared with the BD+APEC group, LB+APEC significantly upregulated *ZO-1* and *ZO-2* expression (*P* < 0.05). In addition, *ZO-1* expression was higher in the HB+APEC group than in the LB+APEC group (*P* < 0.05) (**Figure 3A**). At 42 d, *ZO-1* expression was significantly downregulated in the BD+APEC group compared with the BD+Saline group (*P* < 0.05). Compared with the BD+APEC group, LB+APEC significantly upregulated *ZO-1*, *ZO-2*, and *Claudin-1* expression, whereas HB+APEC significantly upregulated *ZO-2* expression (*P* < 0.05and*P* < 0.01). The expression levels of *ZO-1*, *ZO-2*, and *Claudin-1* were higher in the LB+APEC group than in the HB+APEC group (*P* < 0.05) (**Figure 3B**).

**Figure 3:**
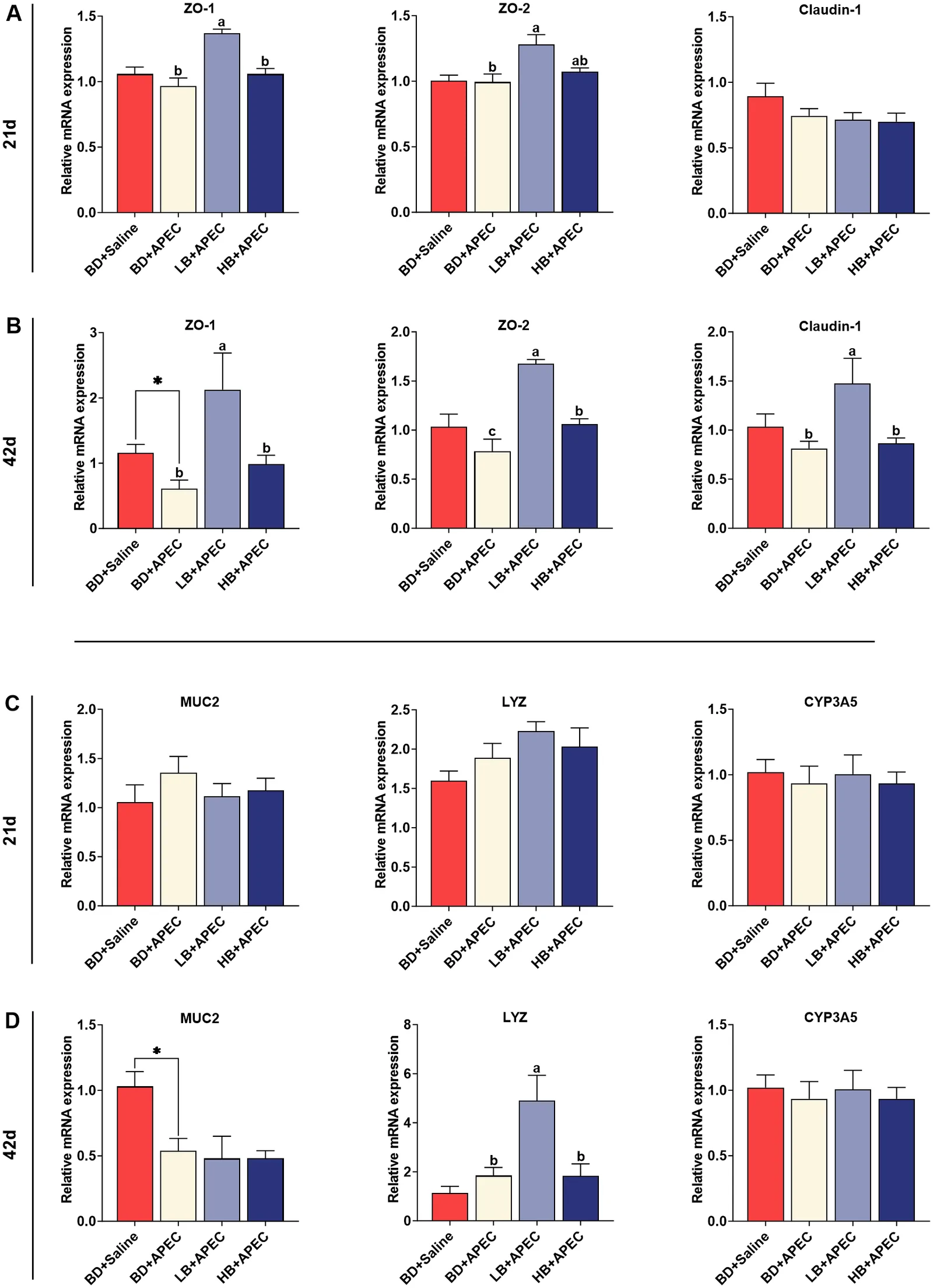
Effects of dietary baicalin supplementation on ileal physical and chemical barrier-related gene expression in broilers after avian pathogenic *Escherichia coli* (APEC) challenge. The relative mRNA expression levels of **(A)** physical barrier-related genes (*ZO-1*, *ZO-2*, and *Claudin-1*) at d 21, **(B)** physical barrier-related genes (*ZO-1*, *ZO-2*, and *Claudin-1*) at d 42, **(C)** chemical barrier-related genes (*MUC2*, *LYZ*, and *CYP3A5*) at d 21, and **(D)** chemical barrier-related genes (*MUC2*, *LYZ*, and *CYP3A5*) at d 42. BD+Saline, broilers fed the basal diet and orally administered saline; BD+APEC, broilers fed the basal diet and orally challenged with APEC; LB+APEC, broilers fed the basal diet supplemented with 150 mg/kg baicalin and orally challenged with APEC; HB+APEC, broilers fed the basal diet supplemented with 300 mg/kg baicalin and orally challenged with APEC.*P < 0.05 and **P < 0.01 indicate differences between the BD+Saline and BD+APEC groups; ns indicates no significant difference. Different lowercase letters indicate significant differences among the BD+APEC, LB+APEC, and HB+APEC groups (*P* < 0.05).

### 3.6 Ileal chemical barrier

At 21 d, the mRNA expression levels of *MUC2*, *LYZ*, and *CYP3A5* showed no significant changes in the ileal tissues of broiler chickens among the treatment groups (**Figure 3C**). At 42 d, MUC2 mRNA expression was significantly downregulated in the BD+APEC group compared with the BD+Saline group (*P* < 0.05). Compared with the BD+APEC group, *LYZ* mRNA expression was significantly upregulated in the LB+APEC group (*P* < 0.05), whereas *MUC2* and *CYP3A5* expression were not significantly altered by baicalin supplementation (**Figure 3D**).

### 3.7 Ileal inflammatory response

At 21 d, *NF-κB* and *TNF-α* mRNA expression levels were significantly upregulated in the BD+APEC group compared with the BD+Saline group (*P* < 0.01), whereas *IL-1β*, *IL-6*, and *IL-8* expression levels were not significantly changed. Compared with the BD+APEC group, both LB+APEC and HB+APEC significantly downregulated *NF-κB*, *TNF-α*, *IL-6*, and *IL-8* expression (*P* < 0.05). *IL-1β* expression was significantly downregulated only in the HB+APEC group (*P* < 0.05) (**Figure 4A**). At 42 d, *NF-κB* and *TNF-α* mRNA expression levels were significantly upregulated in the BD+APEC group compared with the BD+Saline group (*P* < 0.01). Compared with the BD+APEC group, LB+APEC significantly downregulated *NF-κB* expression but upregulated *IL-8* expression (*P* < 0.05). HB+APEC significantly downregulated *NF-κB* and *TNF-α* expression (*P* < 0.05). Compared with the LB+APEC group, the HB+APEC group showed lower *TNF-α*, *IL-6*, and *IL-8* expression levels (*P* < 0.05) (**Figure 4B**).

**Figure 4:**
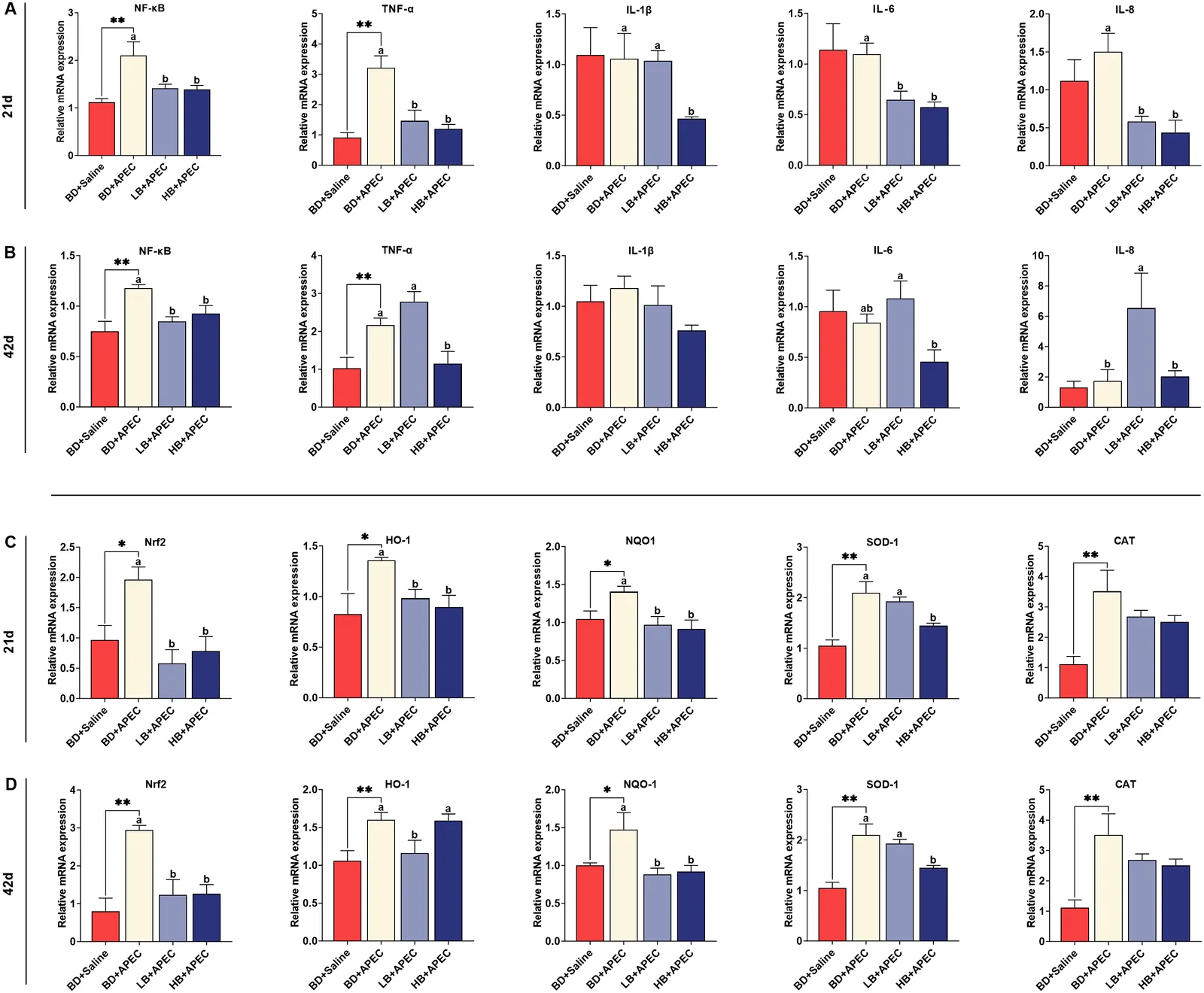
Effects of dietary baicalin supplementation on ileal inflammatory and Nrf2-related antioxidant gene expression in broilers after avian pathogenic *Escherichia coli* (APEC) challenge. The relative mRNA expression levels of **(A)** inflammatory genes (*NF-κB*, *TNF-α*, *IL-1β*, *IL-6*, and *IL-8*) at d 21, **(B)** inflammatory genes (*NF-κB*, *TNF-α*, *IL-1β*, *IL-6*, and *IL-8*) at d 42, **(C)** Nrf2-related antioxidant genes (*Nrf2*, *HO-1*, *NQO1*, *SOD1*, and *CAT*) at d 21, and **(D)** Nrf2-related antioxidant genes (*Nrf2*, *HO-1*, *NQO1*, *SOD1*, and *CAT*) at d 42. BD+Saline, broilers fed the basal diet and orally administered saline; BD+APEC, broilers fed the basal diet and orally challenged with APEC; LB+APEC, broilers fed the basal diet supplemented with 150 mg/kg baicalin and orally challenged with APEC; HB+APEC, broilers fed the basal diet supplemented with 300 mg/kg baicalin and orally challenged with APEC. *P < 0.05 and **P < 0.01 indicate differences between the BD+Saline and BD+APEC groups; ns indicates no significant difference. Different lowercase letters indicate significant differences among the BD+APEC, LB+APEC, and HB+APEC groups (*P* < 0.05).

### 3.8 Nrf2-related antioxidant gene expression

At 21 d, compared with the BD+Saline group, the mRNA expression levels of *Nrf2*, *HO-1*, and *NQO1* were significantly upregulated in the BD+APEC group (*P* < 0.05), while *SOD1* and *CAT* expression levels were significantly upregulated (*P* < 0.01) (**Figure 4C**). Compared with the BD+APEC group, the mRNA expression levels of *Nrf2* (*P* < 0.01), *HO-1* (*P* < 0.05), and *NQO1* (*P* < 0.05) were significantly downregulated in both the LB+APEC and HB+APEC groups. In addition, *SOD1* expression was significantly downregulated in the HB+APEC group (*P* < 0.05), whereas *CAT* expression showed no significant change (**Figure 4C**). At 42 d, compared with the BD+Saline group, the mRNA expression levels of *Nrf2*, *HO-1*, and *NQO1* were significantly upregulated in the BD+APEC group (*P* < 0.05), while *SOD1* and *CAT* expression levels were significantly upregulated (*P* < 0.01) (**Figure 4D**). Compared with the BD+APEC group, the mRNA expression levels of *Nrf2* and *NQO1* were significantly downregulated in both the LB+APEC and HB+APEC groups (*P* < 0.05), *HO-1* expression was significantly downregulated in the LB+APEC group (*P* < 0.05), and *SOD1* expression was significantly downregulated in the HB+APEC group (*P* < 0.01) (**Figure 4D**).

### 3.9 Intestinal biological barrier

At d 21, the BD+APEC group showed lower Observed species, ACE, Chao1, Shannon, and Simpson indices than the BD+Saline group (*P* < 0.05; **Figure 5A**). The ileal microbial communities of the BD+Saline and BD+APEC groups were separated in the ordination analysis (**Figure 5B**). The stacked bar plots showed that Bacillota, Pseudomonadota, and Bacteroidota were the predominant phyla, followed by Actinomycetota and Campylobacterota; 15 major families and 20 genera with relative abundance >1% were observed (**Figures 5D–F**). Compared with the BD+Saline group, APEC challenge increased the relative abundances of *Candidatus Arthromitus* and *Helicobacter* and decreased those of *Asinibacterium*, *Afipia*, *Labrys*, *Hyphomicrobium*, *Pelomonas*, *Shinella*, *Microvirga*, *Noviherbaspirillum*, *Pandoraea*, and *Streptococcus* (*P* < 0.05; **Figure 5J**). Compared with the BD+APEC group, the HB+APEC group showed higher Observed species, ACE, Chao1, and Shannon indices (*P* < 0.05; **Figure 5A**). Differences in microbial community structure were also observed among the APEC-challenged groups (**Figure 5C**). In these groups, Bacillota, Pseudomonadota, Actinomycetota, Campylobacterota, and Bacteroidota were the major phyla, with 15 major families and 18 genera exceeding 1% relative abundance (**Figures 5G–I**). Relative to the BD+APEC group, the LB+APEC group showed higher relative abundances of *Corynebacterium*, *Weissella*, *Aerococcus*, *Kurthia*, and *Glutamicibacter* and lower relative abundances of *Shinella*, *Dyella*, *Acidovorax*, *Campylobacter*, and *Microvirga* (*P* < 0.05; **Figure 5K**).

**Figure 5:**
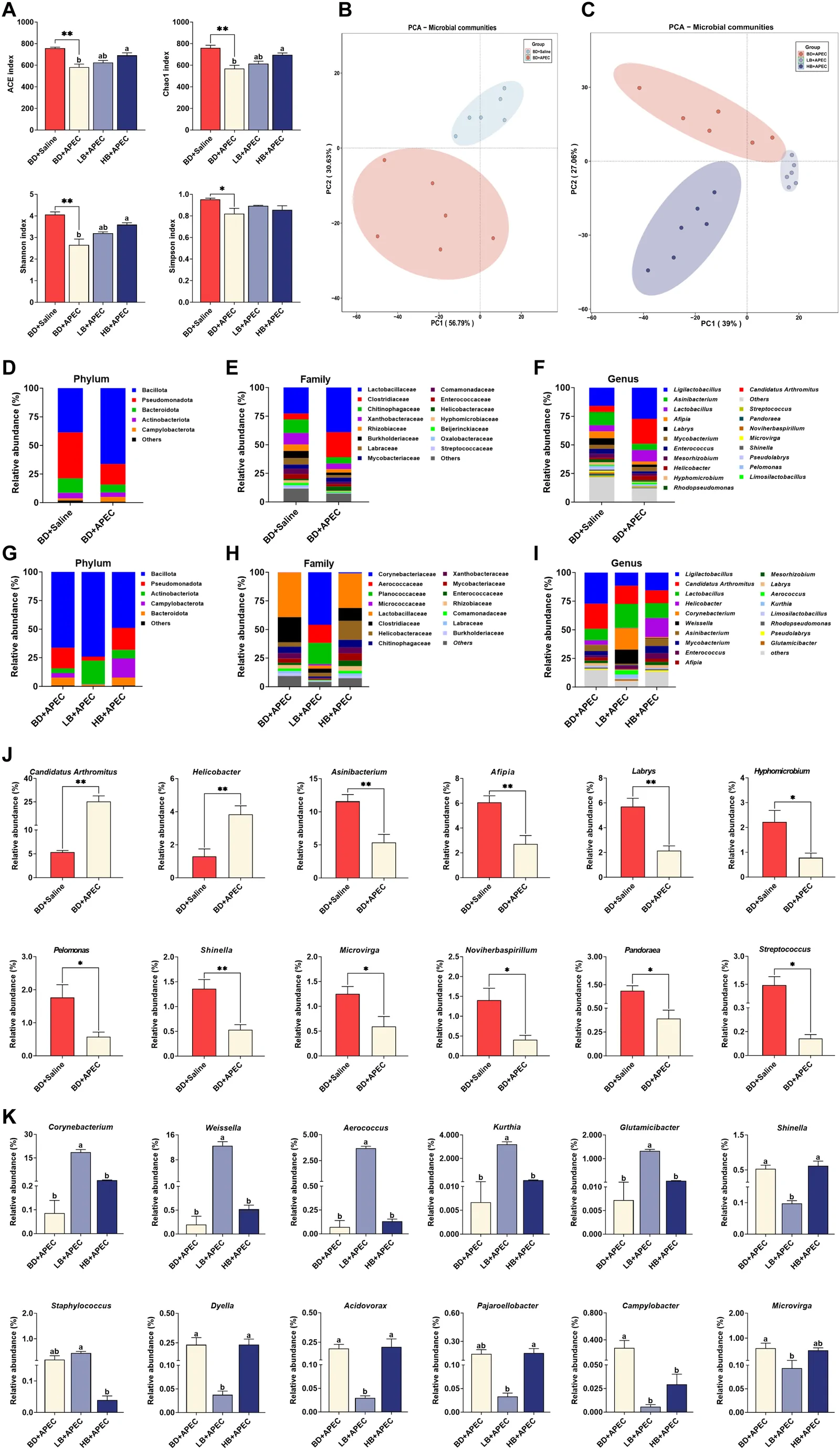
Effects of dietary baicalin supplementation on the ileal microbiota of APEC-challenged broilers at d 21. **(A)** Alpha-diversity indices of the ileal microbiota. **(B)** Ordination of microbial communities between the BD+Saline and BD+APEC groups. **(C)** Ordination of microbial communities among the BD+APEC, LB+APEC, and HB+APEC groups. Taxonomic composition at the phylum, family, and genus levels between the BD+Saline and BD+APEC groups is shown in **(D–F)**, respectively, and among the BD+APEC, LB+APEC, and HB+APEC groups in **(G–I)**, respectively. **(J)** Differentially abundant bacterial genera between the BD+Saline and BD+APEC groups. **(K)** Differentially abundant bacterial genera among the BD+APEC, LB+APEC, and HB+APEC groups. *P < 0.05 and **P < 0.01 indicate differences between the BD+Saline and BD+APEC groups. Different lowercase letters indicate significant differences among the BD+APEC, LB+APEC, and HB+APEC groups (*P* < 0.05).

At d 42, ACE and Chao1 indices were lower in the BD+APEC group than in the BD+Saline group (*P* < 0.05), whereas Shannon and Simpson indices were unchanged (**Figure 6A**). Differences in ileal microbial community structure were observed between the BD+Saline and BD+APEC groups (**Figure 6B**). The stacked bar plots showed that Bacillota was the predominant phylum, followed mainly by Pseudomonadota, Thermodesulfobacteriota, and Bacteroidota; nine major families and nine genera with relative abundance >1% were observed (**Figures 6D–F**). Compared with the BD+Saline group, APEC challenge increased the relative abundances of *Serratia*, *Erwinia*, and *Pygmaiobacter* (*P* < 0.05; **Figure 6J**). Compared with the BD+APEC group, the LB+APEC group showed higher ACE and Chao1 indices, whereas the HB+APEC group showed higher Chao1 and Shannon indices (*P* < 0.05; **Figure 6A**). Differences in microbial community structure were also observed among the APEC-challenged groups (**Figure 6C**). In these groups, Bacillota, Pseudomonadota, and Actinomycetota were the major phyla, followed by Bacteroidota, Thermodesulfobacteriota, Fusobacteriota, and Campylobacterota; 20 major families and 19 genera exceeded 1% relative abundance (**Figures 6G–I**). Baicalin supplementation altered several bacterial genera. In particular, *Corynebacterium* and *Luteitalea* were increased in the LB+APEC group, whereas *Ligilactobacillus*, *Lactobacillus*, *Limosilactobacillus*, *Klebsiella*, and *Citrobacter* were decreased to varying extents in the baicalin-supplemented groups (*P* < 0.05; **Figure 6K**).

**Figure 6:**
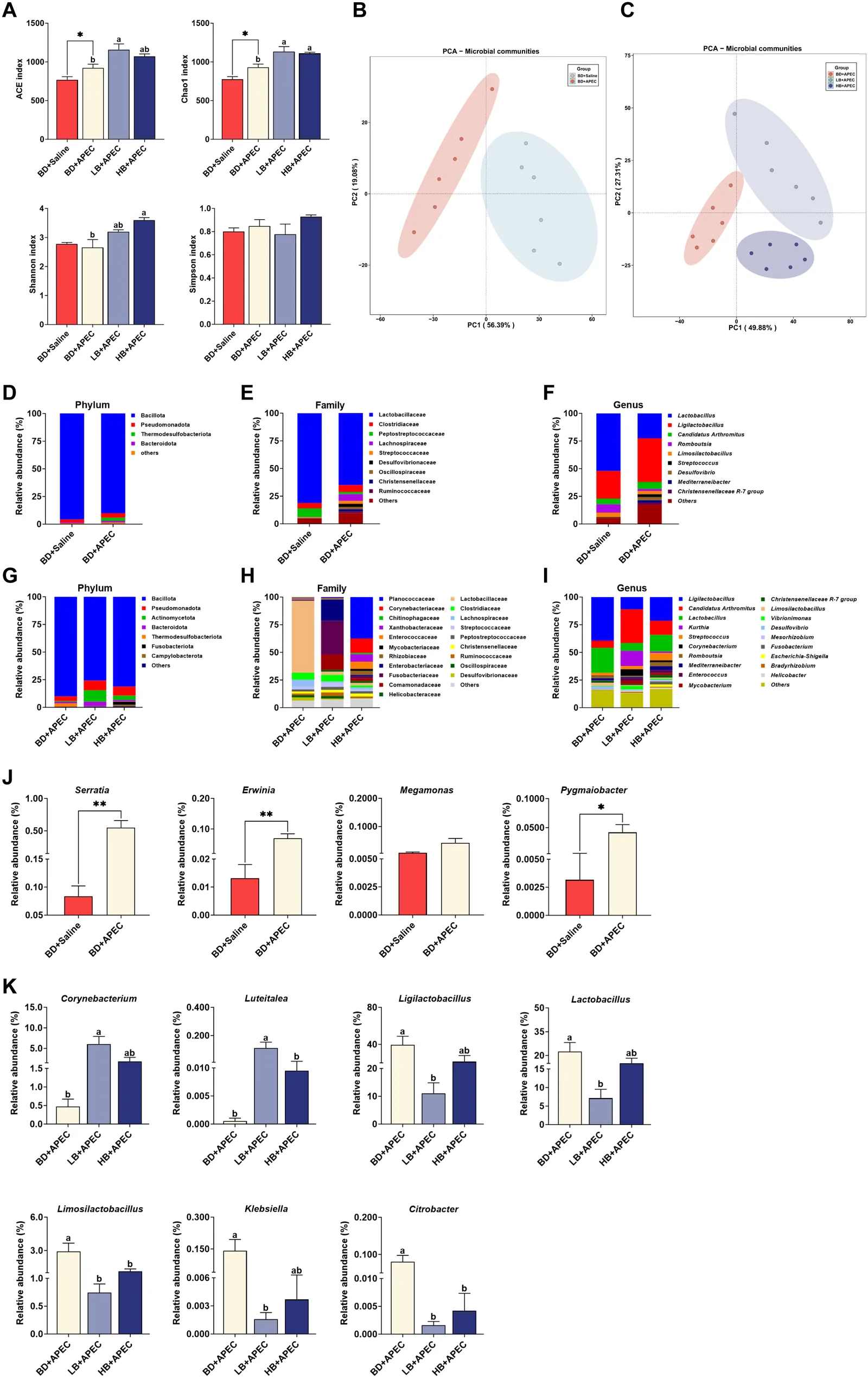
Effects of dietary baicalin supplementation on the ileal microbiota of APEC-challenged broilers at d 42. **(A)** Alpha-diversity indices of the ileal microbiota. **(B)** Ordination of microbial communities between the BD+Saline and BD+APEC groups. **(C)** Ordination of microbial communities among the BD+APEC, LB+APEC, and HB+APEC groups. Taxonomic composition at the phylum, family, and genus levels between the BD+Saline and BD+APEC groups is shown in **(D–F)**, respectively, and among the BD+APEC, LB+APEC, and HB+APEC groups in **(G–I)**, respectively. **(J)** Differentially abundant bacterial genera between the BD+Saline and BD+APEC groups. **(K)** Differentially abundant bacterial genera among the BD+APEC, LB+APEC, and HB+APEC groups. *P < 0.05 and **P < 0.01 indicate differences between the BD+Saline and BD+APEC groups. Different lowercase letters indicate significant differences among the BD+APEC, LB+APEC, and HB+APEC groups (*P* < 0.05).

### 3.10 Ileal transcriptome

At d 21, PCA showed separation between the BD+Saline and BD+APEC groups (**Figure 7A**). A total of 1,229 DEGs were identified, including 847 upregulated and 382 downregulated genes in the BD+APEC group (**Figure 7B**). GO analysis mainly highlighted digestion and nutrient metabolism, whereas KEGG analysis showed enrichment in glutathione metabolism, cell adhesion molecules, arachidonic acid metabolism, antigen processing and presentation, and drug metabolism–cytochrome P450 (**Figures 7C–E**). A PPI network was constructed using DEGs from the significantly enriched pathways (**Figure 7F**).

**Figure 7:**
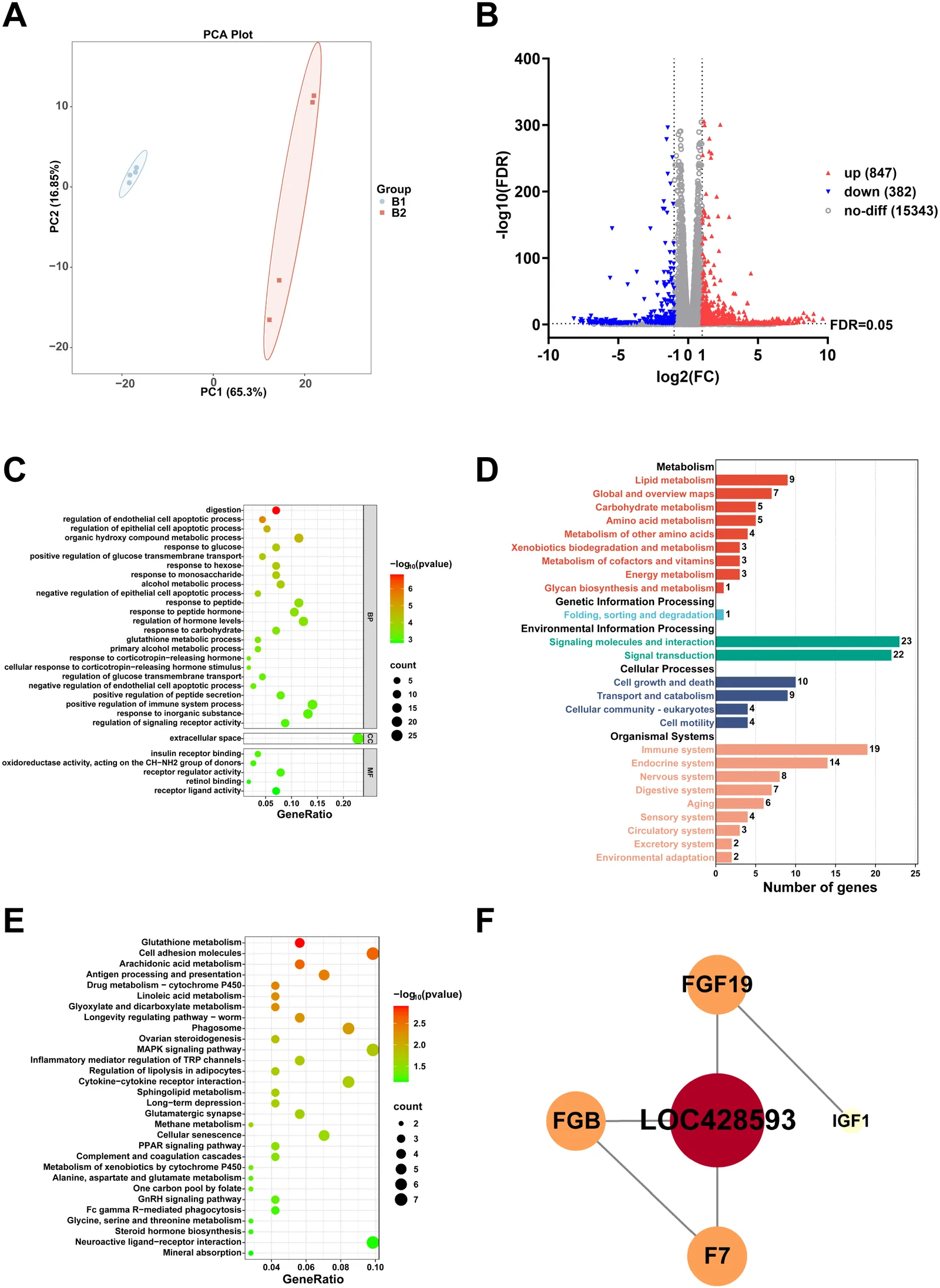
Transcriptomic analysis of ileal tissue between the BD+Saline and BD+APEC groups at d 21. **(A)** Principal component analysis (PCA). **(B)** Volcano plot of differentially expressed genes (DEGs). **(C)** Gene Ontology (GO) enrichment analysis of DEGs, including biological process (BP), cellular component (CC), and molecular function (MF). **(D)** Kyoto Encyclopedia of Genes and Genomes (KEGG) pathway classification of DEGs. **(E)** KEGG pathway enrichment analysis of DEGs. **(F)** Protein-protein interaction (PPI) network of DEGs from significantly enriched pathways. BD+Saline, broilers fed the basal diet and orally administered saline; BD+APEC, broilers fed the basal diet and orally challenged with avian pathogenic *Escherichia coli* (APEC).

At d 21, PCA also showed separation between the BD+APEC and LB+APEC groups (**Figure 8A**). A total of 1,493 DEGs were identified, with 379 upregulated and 1,114 downregulated genes following baicalin supplementation (**Figure 8B**). GO analysis highlighted muscle-related processes and lymphocyte-mediated immune responses, while KEGG analysis identified enrichment in cytoskeleton in muscle cells, phagosome, neuroactive ligand–receptor interaction, and cAMP signaling (**Figures 8C–E**). PPI analysis identified *NPY* and *MYL10* as hub genes; *NPY* was upregulated and *MYL10* was downregulated following baicalin supplementation (**Figure 8F**).

**Figure 8:**
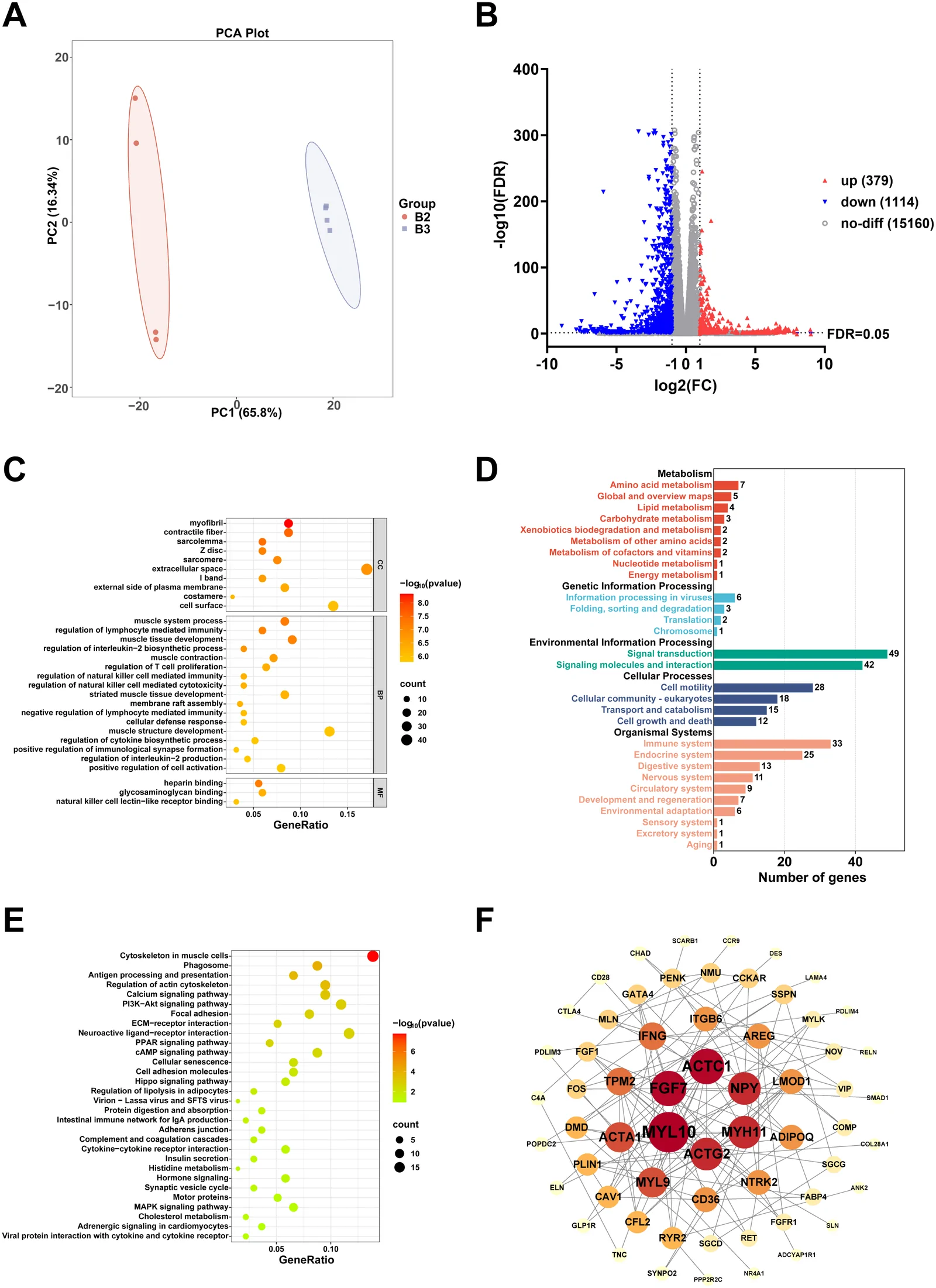
Transcriptomic analysis of ileal tissue between the BD+APEC and LB+APEC groups at d 21. **(A)** Principal component analysis (PCA). **(B)** Volcano plot of differentially expressed genes (DEGs). **(C)** Gene Ontology (GO) enrichment analysis of DEGs, including biological process (BP), cellular component (CC), and molecular function (MF). **(D)** Kyoto Encyclopedia of Genes and Genomes (KEGG) pathway classification of DEGs. **(E)** KEGG pathway enrichment analysis of DEGs. **(F)** Protein-protein interaction (PPI) network of DEGs from significantly enriched pathways, with neuropeptide Y (*NPY*) and myosin light chain 10 (*MYL10*) identified as hub genes. BD+APEC, broilers fed the basal diet and orally challenged with avian pathogenic *Escherichia coli* (APEC); LB+APEC, broilers fed the basal diet supplemented with 150 mg/kg baicalin and orally challenged with APEC.

At d 42, PCA showed separation between the BD+Saline and BD+APEC groups (**Figure 9A**). A total of 1,940 DEGs were identified, including 927 upregulated and 1,013 downregulated genes in the BD+APEC group (**Figure 9B**). GO analysis mainly highlighted defense and immune-related processes, whereas KEGG analysis showed enrichment in cytokine–cytokine receptor interaction and drug metabolism–cytochrome P450 (**Figures 9C–E**). PPI analysis identified *IL-10* and *IFN-γ* as hub genes, both of which were downregulated following APEC challenge (**Figure 9F**).

**Figure 9:**
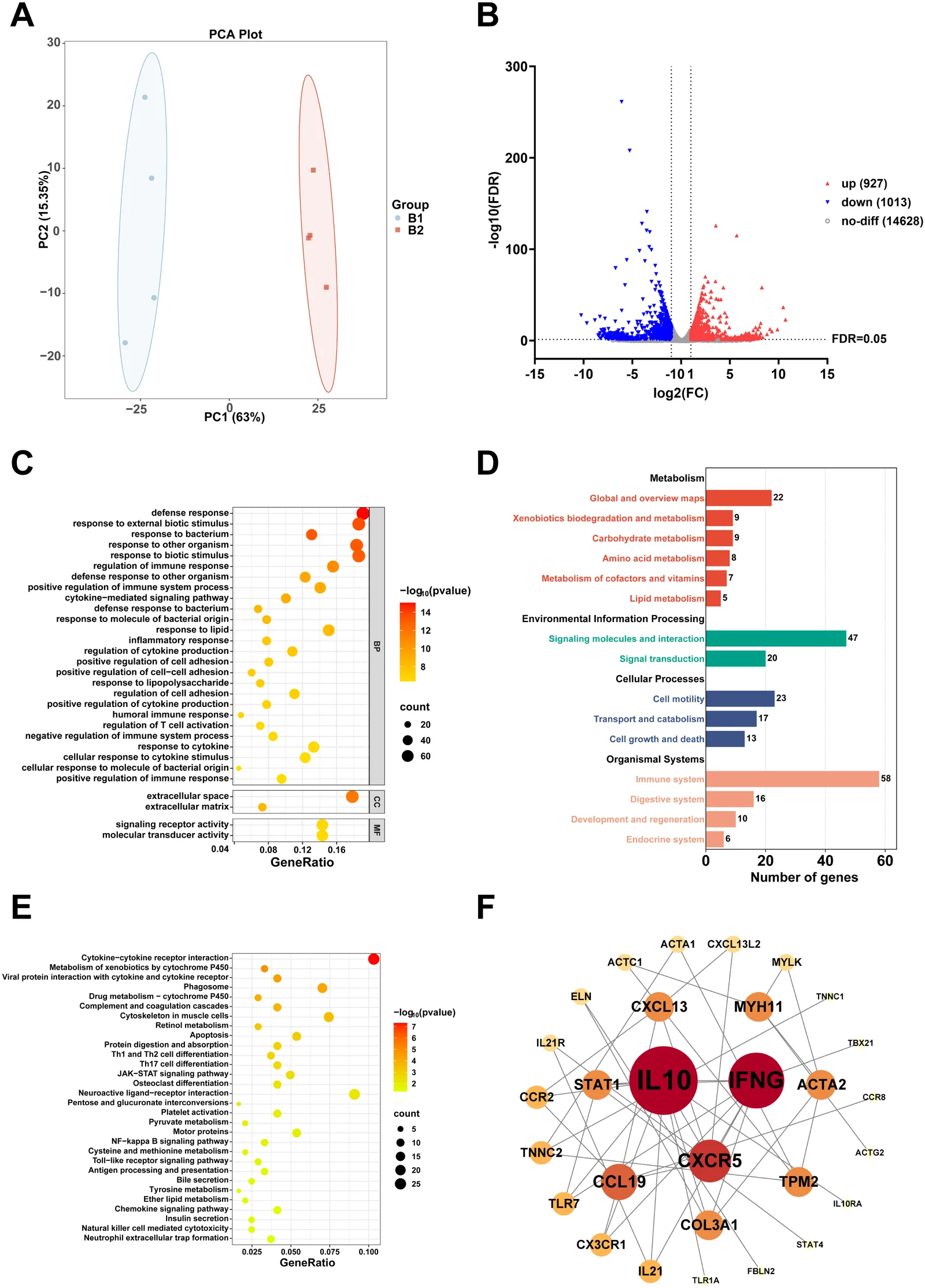
Transcriptomic analysis of ileal tissue between the BD+Saline and BD+APEC groups at d 42. **(A)** Principal component analysis (PCA). **(B)** Volcano plot of differentially expressed genes (DEGs). **(C)** Gene Ontology (GO) enrichment analysis of DEGs, including biological process (BP), cellular component (CC), and molecular function (MF). **(D)** Kyoto Encyclopedia of Genes and Genomes (KEGG) pathway classification of DEGs. **(E)** KEGG pathway enrichment analysis of DEGs. **(F)** Protein-protein interaction (PPI) network of DEGs from significantly enriched pathways, with interleukin-10 (*IL-10*) and interferon-gamma (*IFN-γ*) identified as hub genes. BD+Saline, broilers fed the basal diet and orally administered saline; BD+APEC, broilers fed the basal diet and orally challenged with avian pathogenic *Escherichia coli* (APEC).

At d 42, PCA showed separation between the BD+APEC and LB+APEC groups (**Figure 10A**). A total of 1,456 DEGs were identified, with 869 upregulated and 587 downregulated genes following baicalin supplementation (**Figure 10B**). GO analysis highlighted synaptic signaling and G protein-coupled receptor activity, while KEGG analysis showed enrichment in neuroactive ligand–receptor interaction and cAMP signaling (**Figures 10C–E**). PPI analysis identified *NPY* and *AGTR1* as hub genes; *NPY* was upregulated and *AGTR1* was downregulated following baicalin supplementation (**Figure 10F**).

**Figure 10:**
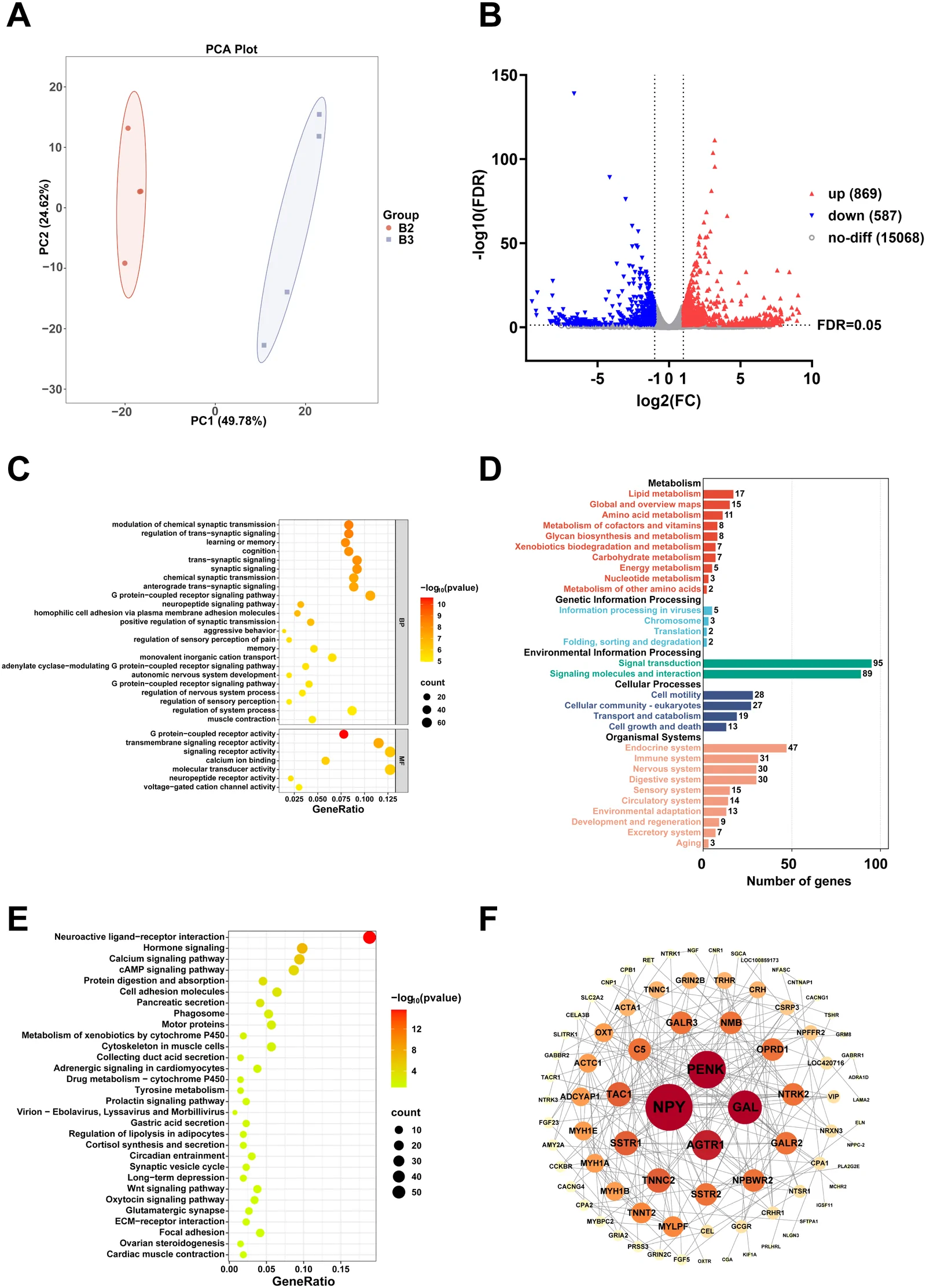
Transcriptomic analysis of ileal tissue between the BD+APEC and LB+APEC groups at d 42. **(A)** Principal component analysis (PCA). **(B)** Volcano plot of differentially expressed genes (DEGs). **(C)** Gene Ontology (GO) enrichment analysis of DEGs, including biological process (BP), cellular component (CC), and molecular function (MF). **(D)** Kyoto Encyclopedia of Genes and Genomes (KEGG) pathway classification of DEGs. **(E)** KEGG pathway enrichment analysis of DEGs. **(F)** Protein-protein interaction (PPI) network of DEGs from significantly enriched pathways, with neuropeptide Y (*NPY*) and angiotensin II receptor type 1 (*AGTR1*) identified as hub genes. BD+APEC, broilers fed the basal diet and orally challenged with avian pathogenic *Escherichia coli* (APEC); LB+APEC, broilers fed the basal diet supplemented with 150 mg/kg baicalin and orally challenged with APEC.

### 3.11 qRT-PCR analysis of selected transcriptome-related genes

To further examine selected transcriptome-related genes, *NPY*, *NPY1R*, *AGTR1*, *IL-10*, *IFN-γ*, and *MYL10* were quantified by qRT-PCR at d 21 and d 42. At d 21, APEC challenge decreased *NPY* expression and increased *AGTR1* and *MYL10* expression. Baicalin supplementation increased *NPY*, *NPY1R*, and *IL-10* expression and decreased *IFN-γ* and *MYL10* expression to varying extents (*P* < 0.05; **Figure 11A**). At d 42, APEC challenge decreased *NPY* and *NPY1R* expression and increased *IFN-γ* and *MYL10* expression. Baicalin supplementation increased *NPY*, *NPY1R*, and *IL-10* expression and decreased *AGTR1*, *IFN-γ*, and *MYL10* expression (*P* < 0.05; **Figure 11B**).

**Figure 11:**
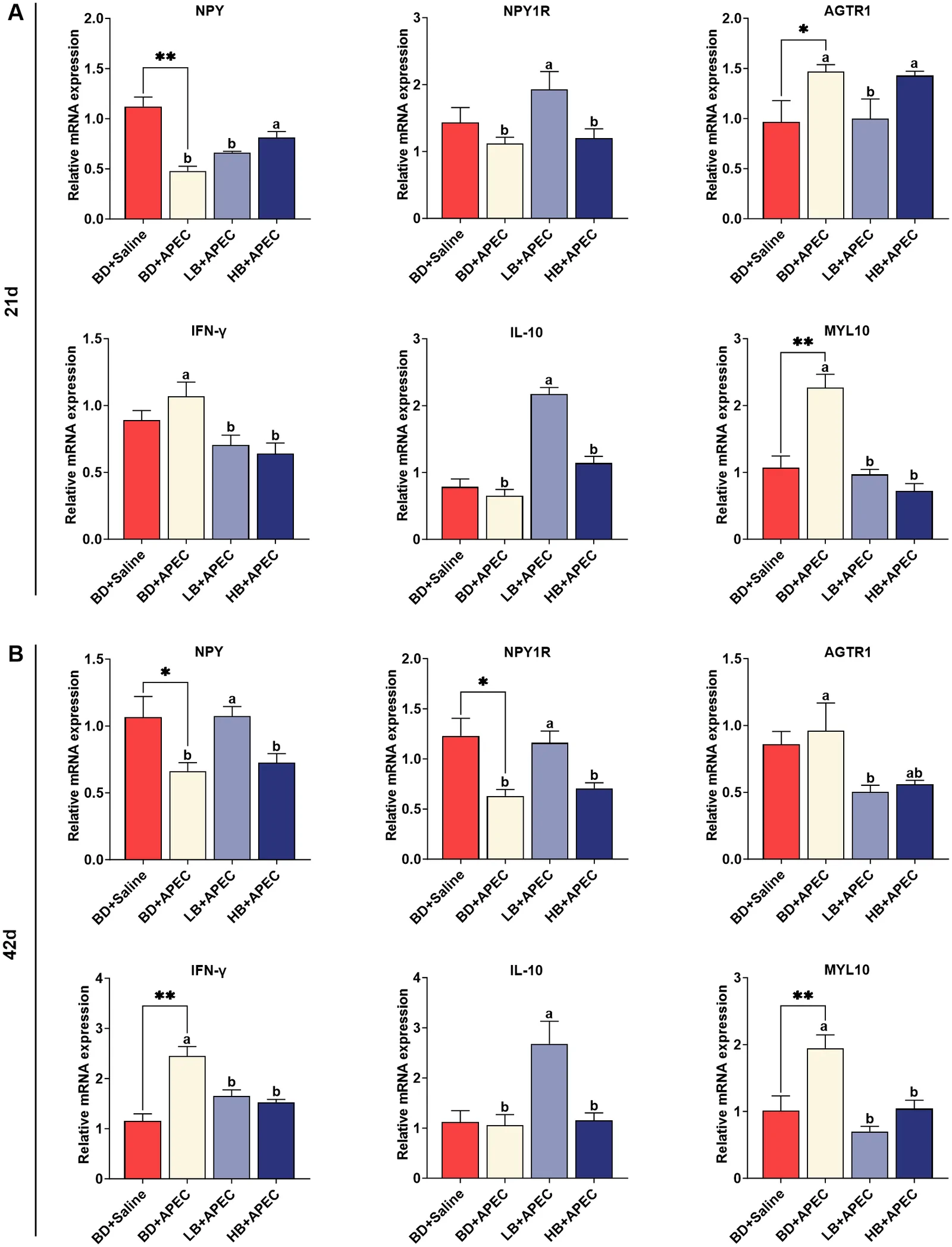
qRT-PCR analysis of selected transcriptome-related genes in the ileum of broilers after avian pathogenic *Escherichia coli* (APEC) challenge. The relative mRNA expression levels of *NPY*, *NPY1R*, *AGTR1*, *IFN-γ*, *IL-10*, and *MYL10* at **(A)** d 21 and **(B)** d 42. BD+Saline, broilers fed the basal diet and orally administered saline; BD+APEC, broilers fed the basal diet and orally challenged with APEC; LB+APEC, broilers fed the basal diet supplemented with 150 mg/kg baicalin and orally challenged with APEC; HB+APEC, broilers fed the basal diet supplemented with 300 mg/kg baicalin and orally challenged with APEC. *P < 0.05 and **P < 0.01 indicate differences between the BD+Saline and BD+APEC groups; ns indicates no significant difference. Different lowercase letters indicate significant differences among the BD+APEC, LB+APEC, and HB+APEC groups (*P* < 0.05).

### 3.12 Microbiota-gene correlation analysis

To investigate the specific links mapping the ileal microbial ecology to transcriptional profiles governing barrier function, inflammatory response, oxidative stress, and neuro-immune function, correlation analysis was performed (**Figure 12**). At 21 d, correlation analysis between the BD+Saline and BD+APEC groups identified 17 significant microbiota-gene correlations, including 7 positive and 10 negative correlations (**Figure 12A**). These correlations mainly involved *Candidatus Arthromitus*, *Helicobacter*, *Asinibacterium*, *Afipia*, *Labrys*, *Hyphomicrobium*, *Shinella*, *Microvirga*, *Noviherbaspirillum* and *Azohydromonas*, to transcriptional profiles governing mucosal barrier integrity, inflammatory cascades, oxidative stress, and neuro-immune regulation, including *ZO-1*, *NF-κB*, *TNF-α*, *IL-6*, *Nrf2*, *SOD1*, *NPY*, and *IL-10*. Among the BD+APEC, LB+APEC, and HB+APEC groups at 21 d, altogether, 7 prominent links emerged, dividing into 1 positive and 6 inverse co-alterations (**Figure 12B**). These correlations mainly involved *Weissella*, *Corynebacterium*, *Glutamicibacter*, *Shinella*, *Dyella*, *Acidovorax*, and *Pajaroellobacter*, as well as *MUC2*, *CYP3A5*, *TNF-α*, *Nrf2*, *NPY1R*, and *AGTR1*.

**Figure 12:**
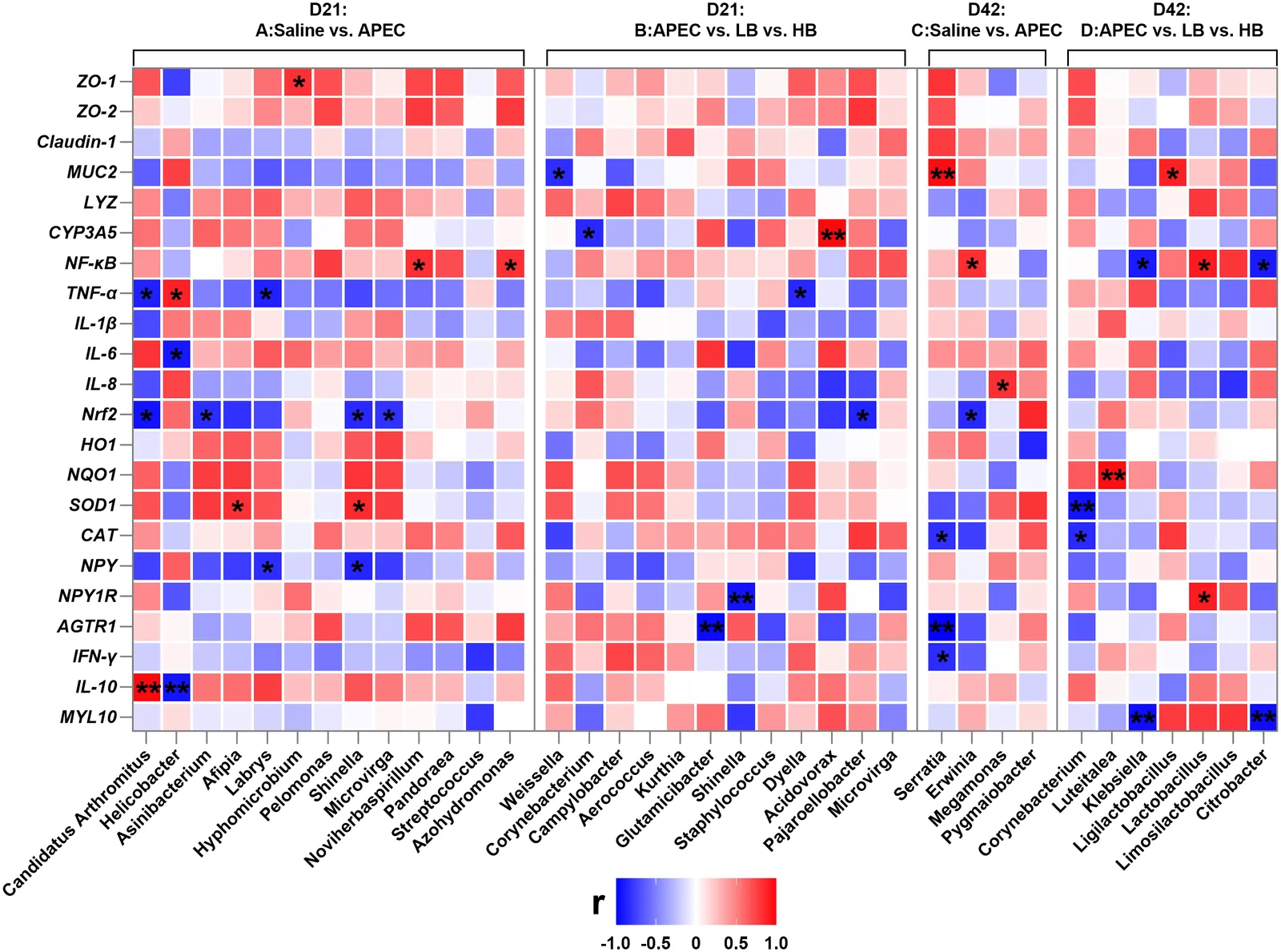
Correlation analysis between the relative abundance of ileal bacterial genera and the expression of genes related to barrier function, inflammatory response, Nrf2-related antioxidant responses, and neuro-immune regulation in broilers. Correlation heatmaps between the relative abundance of ileal microbiota at the genus level and the mRNA expression of selected genes are shown for **(A)** the BD+Saline and BD+APEC groups at d 21, **(B)** the BD+APEC, LB+APEC, and HB+APEC groups at d 21, **(C)** the BD+Saline and BD+APEC groups at d 42, and **(D)** the BD+APEC, LB+APEC, and HB+APEC groups at d 42. Red indicates positive correlations, and blue indicates negative correlations. BD+Saline, broilers fed the basal diet and orally administered saline; BD+APEC, broilers fed the basal diet and orally challenged with avian pathogenic *Escherichia coli* (APEC); LB+APEC, broilers fed the basal diet supplemented with 150 mg/kg baicalin and orally challenged with APEC; HB+APEC, broilers fed the basal diet supplemented with 300 mg/kg baicalin and orally challenged with APEC. *P < 0.05 and **P < 0.01 indicate statistically significant Pearson correlations.

At 42 d, correlation analysis between the BD+Saline and BD+APEC groups identified 7 significant microbiota-gene correlations, including 3 positive and 4 negative correlations (**Figure 12C**). These correlations mainly involved *Serratia*, *Erwinia*, and *Megamonas*, and genes related to barrier function, inflammation, oxidative stress, and neuro-immune regulation, including *MUC2*, *NF-κB*, *IL-8*, *Nrf2*, *CAT*, *AGTR1*, and *IFN-γ*. Among the BD+APEC, LB+APEC, and HB+APEC groups at 42 d, 10 significant correlations were identified, including 4 positive and 6 negative correlations (**Figure 12D**). These correlations mainly involved *Corynebacterium*, *Luteitalea*, *Klebsiella*, *Ligilactobacillus*, *Lactobacillus* and *Citrobacter*, as well as *MUC2*, *NF-κB*, *NQO1*, *SOD1*, *CAT*, *NPY1R* and *MYL10*.

### 3.13 Correlations between selected DEGs and barrier-, inflammation-, and oxidative stress-related genes

Pearson correlation analysis was performed to assess associations between selected neuro-immune-related genes and genes involved in barrier function, inflammatory responses, and Nrf2-related antioxidant responses (**Figure 13**). At d 21, five significant correlations were identified between the BD+Saline and BD+APEC groups, including three positive and two negative correlations (**Figure 13A**), whereas 18 significant correlations were observed among the APEC-challenged groups, including 10 positive and eight negative correlations (**Figure 13B**). These associations mainly involved *NPY*, *NPY1R*, *AGTR1*, *IFN-γ*, *IL-10*, and *MYL10* together with tight junction-, inflammation-, and antioxidant-related genes. At d 42, 10 significant correlations were identified between the BD+Saline and BD+APEC groups, including five positive and five negative correlations (**Figure 13C**), and 17 significant correlations were observed among the APEC-challenged groups, including 12 positive and five negative correlations (**Figure 13D**). These correlations involved neuro-immune-related genes and genes associated with tight junctions, mucus barrier, inflammation, and Nrf2-related antioxidant responses.

**Figure 13:**
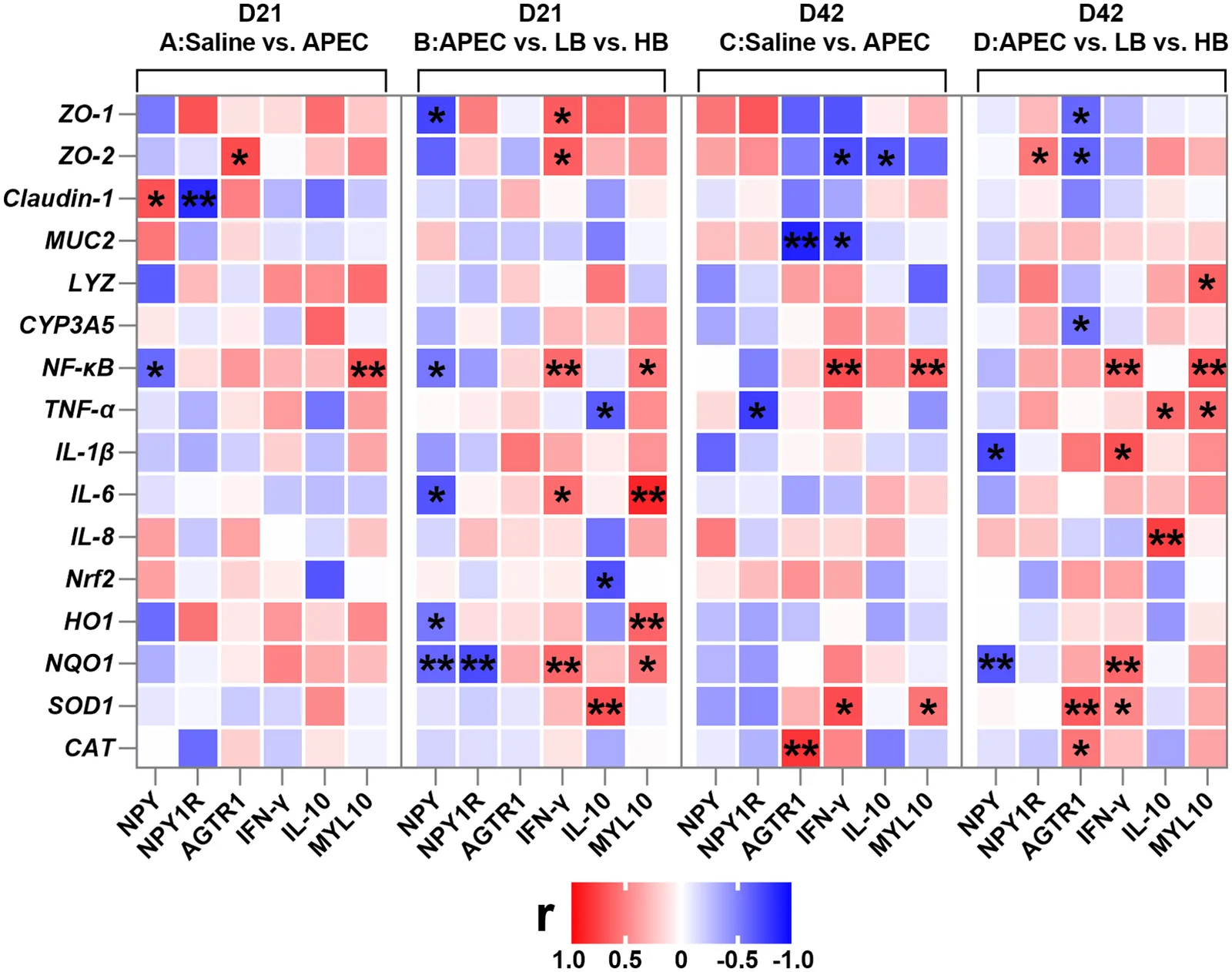
Correlation analysis between selected neuro-immune-related genes and genes related to barrier function, inflammatory response, and Nrf2-related antioxidant responses in the ileum of broilers. Correlation heatmaps between the mRNA expression of selected neuro-immune-related genes (*NPY*, *NPY1R*, *AGTR1*, *IFN-γ*, *IL-10*, and *MYL10*) and barrier-, inflammation-, and antioxidant-related genes are shown for **(A)** the BD+Saline and BD+APEC groups at d 21, **(B)** the BD+APEC, LB+APEC, and HB+APEC groups at d 21, **(C)** the BD+Saline and BD+APEC groups at d 42, and **(D)** the BD+APEC, LB+APEC, and HB+APEC groups at d 42. Red indicates positive correlations, and blue indicates negative correlations. BD+Saline, broilers fed the basal diet and orally administered saline; BD+APEC, broilers fed the basal diet and orally challenged with avian pathogenic *Escherichia coli* (APEC); LB+APEC, broilers fed the basal diet supplemented with 150 mg/kg baicalin and orally challenged with APEC; HB+APEC, broilers fed the basal diet supplemented with 300 mg/kg baicalin and orally challenged with APEC. *P < 0.05 and **P < 0.01 indicate statistically significant Pearson correlations.

## 4 Discussion

The present study examined the effects of dietary baicalin on APEC-associated ileal injury in broiler chickens. Although APEC challenge did not impair overall growth performance, it induced clear changes in ileal barrier- and inflammation-related indicators, supporting the presence of intestinal injury. Baicalin partially alleviated these responses, and the following discussion focuses on its effects on intestinal barrier function, inflammation, microbiota, and transcriptional responses.

### 4.1 Growth performance in response to APEC challenge and baicalin supplementation

In the present study, APEC infection did not significantly affect final body weight, ADG, and ADFI, indicating that the challenge had a limited effect on overall growth performance under the present conditions. However, the Mega monas F:G ratio was significantly decreased was significantly decreased in the APEC group during 1–21 d, whereas ADG and ADFI were not significantly affected. This change may reflect a transient alteration in feed utilization during the early challenge stage, but it should be interpreted cautiously because the overall growth response was not impaired (Jindal et al., 2024; Sanjaya et al., 2024; Ling et al., 2025). Although APEC challenge induced clear ileal injury, these intestinal alterations were not accompanied by significant reductions in BW, ADG, or ADFI, suggesting that the local intestinal response did not translate into a marked overall growth response under the present experimental conditions. Compared with the APEC group, ADG in the high-dose baicalin group showed a decreasing trend during 1–42 d, suggesting that the high dose did not provide additional growth-promoting benefits. Overall, baicalin did not directly improve growth performance in the present study, and its protective effects were mainly reflected in intestinal barrier-related indicators rather than growth traits.

### 4.2 Protective effects of baicalin on ileal barrier integrity

Serum DAO, D-LA, and LPS are commonly used as indicators of intestinal barrier permeability (Gilani et al., 2021; Liu H. et al., 2024). APEC challenge increased serum DAO and LPS following the second challenge, supporting impaired intestinal barrier integrity. Baicalin supplementation reduced permeability-related serum markers following the first challenge, suggesting a beneficial effect on intestinal barrier function.

Villus height, crypt depth, and V/C are commonly used to evaluate intestinal mucosal structure and absorptive capacity (Wang J. et al., 2025; Alagbe et al., 2026). whereas goblet cells contribute to mucus production and mucosal defense (Nyström et al., 2021; Du et al., 2024). APEC challenge reduced villus height and V/C, increased crypt depth, and decreased goblet cell numbers in both villi and crypts at d 21 and 42, indicating ileal mucosal injury. Baicalin supplementation increased villus height and V/C and increased goblet cell numbers in APEC-challenged birds, consistent with previous reports that baicalin improves intestinal morphology and barrier function in broilers (Cao et al., 2022; Liu X. et al., 2025). These findings support a protective effect of baicalin on ileal mucosal structure and mucus-associated barrier function during APEC challenge.

Intestinal barrier integrity depends on both epithelial tight junctions and mucosal defense components that limit paracellular permeability and pathogen contact with the epithelium (Lingaraju et al., 2015; Huang et al., 2024). Following the second APEC challenge, reduced *ZO-1* and *MUC2* expression indicated impairment of the physical and mucus barriers. Baicalin supplementation increased the expression of several tight junction-related genes and *LYZ*, supporting its beneficial effect on ileal barrier integrity and antimicrobial defense.

### 4.3 Modulation of ileal inflammatory and Nrf2-related responses by baicalin

Inflammation is a major host response to pathogenic stimulation, and APEC infection can induce the generation of upstream inflammatory signaling molecules within broiler chickens (Shen et al., 2022). *NF-κB* is a core transcription factor regulating inflammatory responses, and its activation can trigger the cascade expression and release of key inflammatory executioners, notably *TNF-α*, *IL-1β*, *IL-6*, and *IL-8* (Liang Y. et al., 2024; Zhou et al., 2024). In the present study, APEC infection upregulated ileal *NF-κB* and *TNF-α* transcript levels at both 21 and 42 d, indicating that APEC challenge increased ileal NF-κB and TNF-α expression at both sampling ages (Tan et al., 2024). Baicalin supplementation downregulated several APEC-induced inflammatory genes, but its effects differed between 21 and 42 d. At 21 d, baicalin broadly reduced the expression of *NF-κB*, *TNF-α*, *IL-6*, and *IL-8*, suggesting an inhibitory effect on the early inflammatory response. At 42 d, this response was more the response differed between supplementation levels: the high-dose group showed stronger suppression of *TNF-α*, *IL-6*, and *IL-8* than the low-dose group, whereas *IL-8* expression was upregulated in the low-dose group. These results suggest that baicalin attenuated APEC-induced ileal inflammation, although its anti-inflammatory effect varied with dose and sampling age.

Oxidative stress is closely associated with inflammatory responses, and APEC infection can induce excessive ROS production (Wang et al., 2024; Yu et al., 2024). To maintain redox balance in the intestinal mucosa, the host activates the *Nrf2* signaling pathway and promotes the expression of downstream antioxidant-related genes, including *HO-1*, *NQO1*, *SOD1*, and *CAT* (Dong et al., 2022; Biabani et al., 2024). In the present study, APEC infection upregulated *Nrf2* and its downstream antioxidant-related genes in the ileum at both 21 and 42 d, suggesting enhanced oxidative stress and a compensatory antioxidant response. Baicalin supplementation downregulated several APEC-induced Nrf2-related genes. At 21 d, baicalin downregulated the expression of *Nrf2*, *HO-1*, and *NQO1*, whereas at 42 d, it mainly downregulated *Nrf2* and *NQO1* expression, with high-dose baicalin also reducing *SOD1* expression. These changes suggest that baicalin may reduce the need for compensatory activation of Nrf2-related antioxidant genes, possibly by alleviating oxidative stress burden under APEC challenge; however, direct oxidative stress markers are needed to further confirm this effect.

### 4.4 Effects of baicalin on ileal microbial composition during APEC challenge

Following the first APEC challenge, ileal microbial diversity and community structure were markedly altered, accompanied by shifts in several immune-associated bacterial genera. The increased abundance of *Candidatus Arthromitus*, more widely recognized as segmented filamentous bacteria, may reflect mucosal immune stimulation after APEC challenge (Meinen-Jochum et al., 2023; Zhang et al., 2025). Its association with *IL-10* suggests a possible relationship with compensatory immune regulation rather than simply indicating microbial disturbance. Conversely, *Helicobacter* is commonly associated with intestinal mucosal inflammation and barrier damage (Wang T. et al., 2025). In the present study, the enrichment of *Helicobacter* was associated with higher *TNF-α* and lower *IL-10* expression, suggesting that this genus was associated with a more pro-inflammatory ileal environment.

Dietary baicalin supplementation partially alleviated the APEC-associated reduction in ileal microbial richness and diversity at d 21. In particular, high-dose baicalin increased the ACE, Chao1, and Shannon indices, suggesting partial recovery of microbial diversity following the first challenge. At the genus level, baicalin altered several APEC-responsive taxa, among which *Weissella* and *Corynebacterium* were further considered because of their potential links with intestinal protection and immune regulation. *Weissella*, a lactic acid bacterium with probiotic potential, may contribute to intestinal protection by producing organic acids and antimicrobial metabolites and by modulating the local microbial environment (Jin et al., 2025). However, its association with *MUC2* in the present study does not establish a direct mucus-promoting role (Liu et al., 2024b). *Corynebacterium* includes metabolically active members with potential immunomodulatory properties, and previous studies have linked this genus to TNF-related inflammatory signaling (Liu T. et al., 2025). These findings indicate that baicalin altered ileal microbial diversity and several bacterial genera following the first APEC challenge, although the functional significance of these taxa requires further validation.

Following the second APEC challenge, ileal microbial richness and community structure were also altered, with increased relative abundances of *Serratia*, *Erwinia*, and *Pygmaiobacter*. Among these taxa, *Serratia* deserves attention because members of this genus can secrete cytotoxins, disrupt epithelial integrity, and promote bacterial translocation through multiple virulence factors (Ochieng et al., 2014; Tuttobene et al., 2023). Therefore, the enrichment of *Serratia* may reflect a shift toward a more injury-associated ileal microbial environment (Boldeanu et al., 2025). In the present study, *Serratia* abundance was associated with higher *MUC2* expression but lower *CAT*, *AGTR1*, and *IFN-γ* expression. These correlations suggest possible relationships between *Serratia* and host barrier, antioxidant, and neuro-immune-related responses, but do not establish causal links (Melhem et al., 2021; Wang et al., 2022). Baicalin supplementation also altered the ileal microbiota following the second challenge, including changes in *Corynebacterium*, *Luteitalea*, *Ligilactobacillus*, *Lactobacillus*, *Limosilactobacillus*, *Klebsiella*, and *Citrobacter*. Together, these findings show that both APEC challenge and baicalin supplementation were associated with changes in ileal microbial community structure, while the biological roles of individual responsive taxa remain to be determined.

### 4.5 Neuro-immune-related transcriptional responses and microbiota–gene associations

Following the first APEC challenge, transcriptomic changes in the ileum involved pathways related to nutrient metabolism, glutathione metabolism, cell adhesion, arachidonic acid metabolism, and antigen processing and presentation. Glutathione and arachidonic acid metabolism are closely related to redox homeostasis and inflammatory regulation (Fu et al., 2021; Zhang et al., 2023). whereas cell adhesion and antigen presentation are important for epithelial integrity and mucosal immune responses (Di Sabatino et al., 2023). These findings indicate that APEC challenge affected metabolic, redox, and immune-related processes in the ileum. Following baicalin supplementation at d 21, neuroactive ligand–receptor interaction and cAMP signaling were among the main enriched pathways, and *NPY* and *MYL10* were identified as candidate hub genes. *NPY* is involved in gastrointestinal neuro–immune regulation and energy metabolism, while cAMP signaling participates in epithelial and immune responses (Zhang et al., 2021; Wei et al., 2022). The concurrent enrichment of neuroactive signaling and cytoskeleton-related processes suggests that these pathways may be involved in the ileal transcriptional response to baicalin. However, the functional roles of *NPY* and *MYL10* in barrier protection require further validation.

Following the second APEC challenge, transcriptomic changes were mainly related to host defense and cytokine signaling. Enrichment of cytokine–cytokine receptor interaction, together with *IL-10* and *IFN-γ* identified as hub genes, highlighted changes in cytokine-related immune regulation (Esteves et al., 2021; Takeuchi, 2022; Branchett et al., 2024). Baicalin-associated DEGs were enriched in neuroactive ligand–receptor interaction and cAMP signaling, with *NPY* and *AGTR1* identified as candidate hub genes. AGTR1-related signaling has been associated with intestinal inflammatory responses (Yang et al., 2023). The repeated enrichment of neuroactive ligand–receptor interaction and cAMP signaling at both sampling ages suggests that neuro-immune-related transcriptional responses may be relevant to baicalin supplementation, although their functional roles require further investigation.

qRT-PCR analysis further highlighted changes in *NPY*, *NPY1R*, *AGTR1*, *IFN-γ*, and *MYL10* following APEC challenge and baicalin supplementation. NPY is involved in gastrointestinal neuro-immune regulation, whereas MYL10 is related to cytoskeletal processes associated with epithelial barrier function (Chandrasekharan et al., 2022; Yang et al., 2023). The coordinated changes in these genes identify neuro-immune-, inflammatory-, and cytoskeleton-related processes as candidate components of the ileal response to baicalin. However, these associations do not establish a direct regulatory mechanism.

At 42 d, APEC infection was characterized by downregulated *NPY* and *NPY1R* expression and upregulated *IFN-γ* and *MYL10* expression, indicating that later ileal injury involved sustained inflammatory activation, disrupted neuropeptide-related signaling, and cytoskeleton-related epithelial stress. The elevation of *IFN-γ* transcript level is highly indicative of an inflammatory epithelial environment, whereas upregulated *MYL10* may indicate cytoskeletal contraction-related barrier stress (Liang et al., 2023). Dietary baicalin supplementation, particularly at the low dose, upregulated *NPY*, *NPY1R*, and *IL-10* expression and downregulated *AGTR1*, *IFN-γ*, and *MYL10* expression. These changes suggest that baicalin shifted the later-stage ileal response toward a less inflammatory and less contractile profile. The decrease in *AGTR1* may also be related to reduced angiotensin II type 1 receptor-associated stress signaling, which has been linked to oxidative stress, inflammation, and intestinal barrier injury. Overall, the selected transcriptomic DEGs suggest that APEC-induced ileal barrier injury during the later stage involved neuro–immune imbalance, inflammatory activation, and cytoskeleton-related epithelial stress, whereas dietary baicalin supplementation mainly acted by restoring NPY/NPY1R-related signaling and reducing AGTR1, IFN-γ, and MYL10-associated stress responses. Correlation results were consistent with these expression changes, but should be regarded as supportive evidence rather than direct mechanistic proof.

Overall, these findings suggest that APEC-induced ileal barrier injury is not only associated with inflammatory cytokine activation but also involves neuro–immune signaling, oxidative stress, mucus barrier alteration, and cytoskeleton-related epithelial stress. The NPY/NPY1R axis may be more closely related to tight junction regulation and inflammatory balance, whereas *AGTR1* may be mainly involved in oxidative stress and mucus barrier responses. The IFN-γ/NF-κB axis appears to reflect sustained inflammatory signaling, and *MYL10* may connect NF-κB/TNF-α-related inflammation with cytoskeletal changes. Therefore, baicalin may alleviate APEC-induced ileal barrier injury by modulating NPY/NPY1R- and AGTR1-related signaling, reducing IFN-γ/NF-κB-mediated inflammatory burden, and weakening MYL10-associated cytoskeleton-related barrier stress.

## 5 Conclusion

Baicalin may alleviate intestinal inflammation and barrier dysfunction by reshaping the microbial community and modulating neuro–immune-related gene expression. In the present study, baicalin mitigated APEC-precipitated ileal barrier compromise in broilers. Baicalin increased the relative abundance of *Corynebacterium* during both growth stages and promoted a substantial expansion in the taxonomic prevalence of flavonoid metabolism-associated genus *Weissella* during the first growth stage. These results suggest that baicalin may help ameliorate intestinal microbial dysbiosis, this protective outcome correlates with substantial remodeling of the gut microbial community profile. Meanwhile, baicalin may regulate NPY/NPY1R and AGTR1-related neuroactive ligand–receptor signaling, attenuate IFN-γ/NF-κB–MYL10-related inflammation–cytoskeletal dysregulation, and modulate neuro–immune-related gene expression. These effects may promote the recovery of intestinal barrier function and help maintain intestinal homeostasis. Overall, our findings support the potential of baicalin as a plant-derived functional feed additive for improving intestinal health in broilers under APEC challenge.

## Data Availability

The data presented in this study are deposited in the NCBI Sequence Read Archive (SRA) repository. The 16S rRNA gene sequencing data are available under BioProject accession number PRJNA1514886 (https://www.ncbi.nlm.nih.gov/bioproject/PRJNA1514886), and the transcriptomic sequencing data are available under BioProject accession number PRJNA1515897 (https://www.ncbi.nlm.nih.gov/bioproject/PRJNA1515897).

## Supplementary material

Supplementary Table 1 Ingredients composition and nutrient specification of basal diet. *Premixed feed for chicks mainly consists of minerals, vitamins, trace elements, and amino acids. Each kilogram of feed provides: Vitamin A, 10,000 IU; Vitamin D3, 4,000 IU; Vitamin E, 30 IU; Vitamin K3, 2.95 mg; Vitamin B1, 7.50 mg; Vitamin B2, 10.0 mg; Vitamin B6, 8.00 mg; Vitamin B12, 0.025 mg; Biotin, 0.25 mg; Folic acid, 1.20 mg; Pantothenic acid, 50.50 mg; Niacin, 65.00 mg; Copper, 8 mg; Iron, 90 mg; Zinc, 75 mg; Manganese, 100 mg; Selenium, 0.30 mg; Iodine, 0.65 mg. *Premixed feed for medium-sized chickens mainly consists of minerals, vitamins, trace elements, and amino acids. Each kilogram of feed provides: Vitamin A, 8000 IU; Vitamin D3, 3500 IU; Vitamin E, 25 IU; Vitamin K3, 2.55 mg; Vitamin B1, 5.50 mg; Vitamin B2, 8.0 mg; Vitamin B6, 6.00 mg; Vitamin B12, 0.020 mg; Biotin, 0.15 mg; Folic acid, 0.80 mg; Pantothenic acid, 45.50 mg; Niacin, 55.00 mg; Copper, 6 mg; Iron, 80 mg; Zinc, 65 mg; Manganese, 70 mg; Selenium, 0.32 mg; Iodine, 0.50 mg.

Supplementary Table 2 Gene-specific primers for real-time quantitative reverse transcription PCR.
