# Supplementary material for: Baicalin alleviates APEC-induced ileal barrier injury in broiler chickens by modulating inflammatory responses, microbiota composition, and neuro-immune-related transcriptional profiles
Source: Front Cell Infect Microbiol. 2026 Oct 20;16:1967613. doi: 10.3389/fcimb.2026.1967613 (PMC13727628; doi:10.3389/fcimb.2026.1967613)
Supplement: Supplementary file 1 [file Table1.docx]

**Supplementary tables**

**Table S1**. Ingredients composition and nutrient specification of basal diet

| Content (%) | d 1-21 | d 22-42 |
| --- | --- | --- |
| Corn | 53.50 | 50.50 |
| Soybean meal | 20.35 | 22.00 |
| Citric acid residue (CAR) | 4.70 |  |
| Corn gluten meal | 5.00 | 5.00 |
| Wheat | 3.00 |  |
| flour |  | 10.00 |
| Expanded soybean | 7.45 | 1.50 |
| Soybean oil | 1.00 | 6.00 |
| Premix* | 5.00 | 5.00 |
| Total | 100.00 | 100.00 |
| Calculated nutrient levels | |  |
| AME (MJ/kg) | 12.47 | 13.80 |
| Crude protein | 21.00 | 18.50 |
| Calcium | 0.92 | 0.80 |
| Total phosphorus | 0.60 | 0.50 |
| Available phosphorus | 0.45 | 0.32 |
| Salt (NaCl) | 0.35 | 0.36 |
| Lysine | 1.30 | 1.12 |
| Methionine | 0.58 | 0.50 |
| Met + Cys | 0.95 | 0.81 |

*Premixed feed for chicks mainly consists of minerals, vitamins, trace elements, and amino acids. Each kilogram of feed provides: Vitamin A, 10,000 IU; Vitamin D3, 4,000 IU; Vitamin E, 30 IU; Vitamin K3, 2.95 mg; Vitamin B1, 7.50 mg; Vitamin B2, 10.0 mg; Vitamin B6, 8.00 mg; Vitamin B12, 0.025 mg; Biotin, 0.25 mg; Folic acid, 1.20 mg; Pantothenic acid, 50.50 mg; Niacin, 65.00 mg; Copper, 8 mg; Iron, 90 mg; Zinc, 75 mg; Manganese, 100 mg; Selenium, 0.30 mg; Iodine, 0.65 mg.

*Premixed feed for medium-sized chickens mainly consists of minerals, vitamins, trace elements, and amino acids. Each kilogram of feed provides: Vitamin A, 8000 IU; Vitamin D3, 3500 IU; Vitamin E, 25 IU; Vitamin K3, 2.55 mg; Vitamin B1, 5.50 mg; Vitamin B2, 8.0 mg; Vitamin B6, 6.00 mg; Vitamin B12, 0.020 mg; Biotin, 0.15 mg; Folic acid, 0.80 mg; Pantothenic acid, 45.50 mg; Niacin, 55.00 mg; Copper, 6 mg; Iron, 80 mg; Zinc, 65 mg; Manganese, 70 mg; Selenium, 0.32 mg; Iodine, 0.50 mg.
