# Supplementary material for: Baicalin alleviates APEC-induced ileal barrier injury in broiler chickens by modulating inflammatory responses, microbiota composition, and neuro-immune-related transcriptional profiles
Source: Front Cell Infect Microbiol. 2026 Oct 20;16:1967613. doi: 10.3389/fcimb.2026.1967613 (PMC13727628; doi:10.3389/fcimb.2026.1967613)
Supplement: Supplementary file 2 [file Table2.docx]

| Genes | Accession number | Primer sequences (5’-3’) | Product size (bp) |
| --- | --- | --- | --- |
| ZO-1 | XM_015278975.1 | F: ACAGCTCATCACAGCCTCCT  R: TGAAGGGCTTACAGGAATGG | 125 |
| ZO-2 | NM_204918.1 | F: CTCTCCCCGGCAGGAAATCG  R: AGTTTGTTCCCTGCTGACGG | 130 |
| Claudin1 | NM_001013611.2 | F: GGTATGGCAACAGAGTGGCT  R: CAGCCAATGAAGAGGGCTGA | 91 |
| MUC2 | XM_040673076.2 | F: CAGGATACGTGTGTGCCCAT  R: GGACGCGTTGCAATCAAAGT | 125 |
| LYZ | NM_205281.2 | F: TCTGGGGAAAGTCTTTGGACG  R: TTTTGCAACACACACCCAGTT | 106 |
| CYP3A5 | NM_001001751.3 | F: CCTGGAATACCGCAAAGGCT  R: TGGGGGTCCATGACAACAAC | 114 |
| NF-κB | NM_001001472.3 | F: CGACCTGGATGTCAGGGACT  R: ATGGCCGTTGTAGTTGGTGG | 148 |
| TNF-α | NM_204267.2 | F: CTGTGGGGCGTGCAGT  R: AGGAAAGCCACTAGGAGCAG | 173 |
| IL-1β | NM_204524.2 | F: GCCTGCAGAAGAAGCCTCG  R: CTCAGGTCGCTGTCAGCAAAG | 172 |
| IL-6 | NM_204628.2 | F: AGGGCCGTTCGCTATTTGAA  R: CAGAGGATTGTGCCCGAACT | 72 |
| IL-8 | NM_205498.2 | F: TGCCAGTGCATTAGCACTCA  R: TTGGCGTCAGCTTCACATCT | 70 |
| Nrf2 | NM_001396902.1 | F: CAGAACCAGATTTGGCACTGTTA  R: TCAATCAAGTTCATGTCCAAAAGG | 189 |
| HO1 | NM_205344.2 | F: CCACGAGTTCAAGCTGGTCA  R: AGCCTCAGGACATGGGATCT | 199 |
| NQO1 | NM_001277619.2 | F: AACCTCTTTCAACCACGCCA  R: AAGCACTCGGGGTTCTTGAG | 153 |
| SOD1 | NM_205064.2 | F: TGATGACCTGGGTAGAGGGG  R: ACAACGGTTAGCACTTGGCT | 104 |
| CAT | NM_001031215.2 | F: CCTGACTATGGCGCACGTAT  R: CAGACACACGAGAAGTGGCT | 106 |
| NPY | NM_205473.2 | F: ATCACCAGGCAGAGATATGGA  R: TCACCACATCGAAGGGTCTT | 120 |
| NPY1R | NM_001031535.2 | F: ACCCACCCCCAGATGTGTAA  R: GCCGAACTCCACCTTATCCC | 107 |
| AGTR1 | NM_205157.4 | F: GCAGGAGAAGAGGGGACTG  R: TGAAGGTCAAACCTGCTCCA | 118 |
| IFN-γ | NM_205149.2 | F: CACTGACAAGTCAAAGCCGC  R: TCAAGTCGTTCATCGGGAGC | 131 |
| IL-10 | NM_001004414.4 | F: AGACGTTCGAGAAGATGGATGA  R: CAGCAGGTACTCCTCGATGTA | 89 |
| MYL10 | NM_001097526.1 | F: GTTGGCAGAGAAAGGACAGGA  R: TCCATGATGGTAAATGCTTCTTGA | 142 |
| GAPDH | NM_204305.2 | F: TTCACCACCATGGAGAAGGC  R: GCACGATGCATTGCTGACAA | 153 |
| β-actin | NM_205518.2 | F: CAGCCAGCCATGGATGATGA  R: GCACGATGCATTGCTGACAA | 150 |
